# A Bipedal Robotic Platform Leveraging Reconfigurable Locomotion Policies for Terrestrial, Aquatic, and Aerial Mobility

**DOI:** 10.3390/biomimetics10060374

**Published:** 2025-06-05

**Authors:** Zijie Sun, Yangmin Li, Long Teng

**Affiliations:** Department of Industrial and Systems Engineering, The Hong Kong Polytechnic University, Hong Kong, China; zijie.sun@connect.polyu.hk

**Keywords:** biorobotics, locomotion policy reconfigurability, multi-terrain robot, robotic jumping, crawling robot, swimming robot

## Abstract

Biological systems can adaptively navigate multi-terrain environments via morphological and behavioral flexibility. While robotic systems increasingly achieve locomotion versatility in one or two domains, integrating terrestrial, aquatic, and aerial mobility into a single platform remains an engineering challenge. This work tackles this by introducing a bipedal robot equipped with a reconfigurable locomotion framework, enabling seven adaptive policies: (1) thrust-assisted jumping, (2) legged crawling, (3) balanced wheeling, (4) tricycle wheeling, (5) paddling-based swimming, (6) air-propelled drifting, and (7) quadcopter flight. Field experiments and indoor statistical tests validated these capabilities. The robot achieved a 3.7-m vertical jump via thrust forces counteracting gravitational forces. A unified paddling mechanism enabled seamless transitions between crawling and swimming modes, allowing amphibious mobility in transitional environments such as riverbanks. The crawling mode demonstrated the traversal on uneven substrates (e.g., medium-density grassland, soft sand, and cobblestones) while generating sufficient push forces for object transport. In contrast, wheeling modes prioritize speed and efficiency on flat terrain. The aquatic locomotion was validated through trials in static water, an open river, and a narrow stream. The flight mode was investigated with the assistance of the jumping mechanism. By bridging terrestrial, aquatic, and aerial locomotion, this platform may have the potential for search-and-rescue and environmental monitoring applications.

## 1. Introduction

The natural world exemplifies unparalleled adaptability in locomotion, where organisms dynamically reconfigure movement strategies to thrive in heterogeneous environments. This flexibility of locomotion enables species to transition seamlessly between disparate terrains and tasks. For example, humans can employ gait modulation to transition between walking, jogging, and fast running, or leverage a burst of legged propulsion to jump efficiently over obstacles. In extreme environments, such as aquatic regions or narrow spaces, humans can also adapt to a quadrupedal motion pattern for swimming and climbing. Flying animals, such as birds, are commonly seen to adopt walking or hopping, especially for foraging and nesting. Some extreme cases, such as the Gannets (*Morus bassanus*), are capable of diving into the sea up to 9 m to prey on fish [1]. Additionally, aquatic creatures like the flying fishes (family *Exocoetidae*) are capable of utilizing rapid underwater motion to initiate gliding in air to escape from predators [2]. Such adaptability confers critical evolutionary advantages for animals, such as an increased chance of survival, enhanced energy efficiency, and environmental resilience. These principles of biological locomotion flexibility directly inspire robotic systems aiming to operate in unstructured, multi-domain environments. By emulating nature’s solutions with policy adaptation, where movement strategies are reconfigured in response to environmental changes, we can transcend the limitations of single-domain robotic platforms.

Here, we refer to the locomotion policy reconfigurability as the capability of a robotic system to reuse its locomotion mechanisms for more than one purpose. According to the literature, following this standard, robots can be classified into two general types: those with locomotion mechanisms for single-purpose mobility (SPM) or those with tunable mobility (TM). In robotics, there is an abundance of systems that rely on the addition of a single mechanism for an added locomotion mode, i.e., the SPM type. Examples include small-to-medium scale jumping robots whose mass ranges from 10 to 1000 g, with features such as the addition of wheel structures for wheeling or rolling [3,4,5], the addition of wheel-like legs (Whegs [6]) for crawling or running [7,8], the addition of fixed wings for aerial gliding after jumping [9,10,11,12,13,14], or addition of an eccentric motor for flat-terrain motion via vibration [15].

Locomotion mechanisms for tunable mobility are commonly seen in those robots that conduct cross-environment motions. For example, there have been terrestrial–aquatic machines capable of conducting crawling (or walking) and swimming in water, such as fish-like machines that use the fins for both swimming and crawling [16,17], amphibious snake-shaped robots [18,19,20] with ground crawling and water-surface swimming capabilities, salamander-inspired robots based on sprawling motion [21,22], and sea turtle-inspired robots [23,24,25]. These machines showed gait adaptability to cross the terrestrial and aquatic environments, yet the locomotion speed and agility of these multi-terrain prototypes are still insufficient. Some aerial–terrestrial platforms have shown the application of appendage repurposing for multimodal locomotion. Sihite et al. [26] proposed an eight-mode robotic platform using the coupled design of four air propellers and wheels, with each wheel-leg structure controlled by two hip servos. The degree of freedom in the hip enables the robot’s transition between multiple modes. Meiri et al. [27] proposed a wheeling-flying design with fewer controlled degrees of freedom. Their prototype coupled the rotation of wheels and propellers without additional motors to enable wheeling. Besides embedding flying propulsion mechanisms in wheeled structures, an inverse way that adapts flight mechanisms for ground locomotion was also proposed, utilized for perching [28], ground locomotion [29], etc. The literature has also demonstrated the design of aerial–aquatic robots that excel in versatile transitioning seamlessly between air and water using the same propulsion mechanism [30,31,32,33], which endows potential in tasks like environmental monitoring, disaster response, and underwater inspection.

The literature provides numerous prototypes across two types of terrains. Yet, scenario and environmental differences such as landscape surface structure, tolerance of space for locomotion, or density of operation medium have caused key challenges in designing a versatile robotic system traversing terrains across land, air, and water. In addition, increased functionalities entail the addition of structural designs and tend to impair the performance of other locomotion modes (e.g., energy efficiency, speed, and obstacle overcoming abilities), considering factors such as increased mass, air drag, and the decreased working space of mechanisms.

In this work, we aim to broaden the achievable robotic operation environment by designing a bipedal robotic platform with reconfigurable locomotion policies. The proposed prototype, weighing around 370 g, is equipped with the following functions, according to the types of terrains or scenarios it encounters: (1) Terrestrial: a. Jumping. b. Crawling. c. Tricycle wheeling d. Balanced wheeling. e. Object transfer; (2) Aquatic: a. Synchronous paddling. b. Air-propelled drifting; (3) Aerial: Quadcopter-mode flight. Table 1 lists the methods used and the dedicated scenarios for these functions.

The locomotion modes are modeled and deployed in a variety of field tests. The tricycle wheeling mode provides the robot with speed and efficiency on flat terrain, while the balanced wheeling mode is designed to initiate seamless transition to jumping mode without the time delay in morphological transformation. The robot uses a synchronous paddling motion (one major motion pattern in this work) for crawling and swimming. In the crawling mode, the bipedal robot showcases the capability to operate under various types of terrain, including cobblestones, sand, grassland, and to crawl over obstacles with a height of its center of mass (CoM) by adaptively tuning the locomotion parameters. Leveraging the crawling motion, the robot also demonstrates the capabilities of moving a heavy object (a wheelable luggage weighing 5 kg) and conducting parcel transfer tasks into target areas. We statistically analyze the push force of the crawling motion with three different paddling frequencies (0.25 Hz, 0.5 Hz, and 1 Hz) on different substrates (ceramic floor, wood board, and foam pad). Utilizing the paddling method, the robot achieves adaptive locomotion by seamlessly transitioning between terrestrial crawling and aquatic propulsion (e.g., from riverbank traversal to open-water swimming), while also demonstrating controlled swimming within narrow waterways.

In the balanced jumping mode, the robot employs a synchronized extension of the two legs and can achieve a vertical jumping height of 3.7 m (with thrust force continuously turned on to compensate for gravity), a record higher than most jumping machines. In extreme cases, where the thrust force fully offsets its gravitational force, the robot conducts the jumping assistive flight, which alleviates the requirement for a constant vertical thrust acceleration to gain height and requires a throttle command at a hovering level to continuously gain height up to 26 m.

## 2. Materials and Methods

### 2.1. Demonstration of the Robot Structure and Locomotion Modes

**Figure 1:**
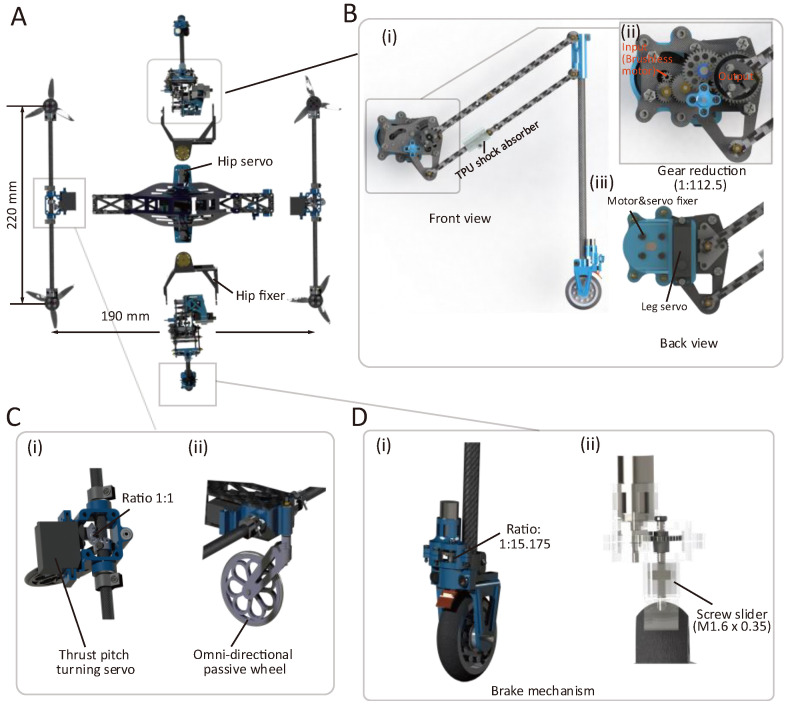
Demonstration of the robot’s structural details. (**A**) Explosive view of the proposed robot. (**B**) Structural details of the leg unit. (**C**) Structural details of the thrust pitch turning servo and the omni-directional passive wheel. (**D**) Structural details of the brake mechanism at the foot.

Figure 1 demonstrates the mechanisms and structural details of the proposed robot. It constitutes four small air thrusters (2.5 inches in diameter) placed with a span of 220 mm × 190 mm (see Figure 1A). These air thrusters enable multiple locomotion strategies, including gravity-offset jumping, tricycle and balanced wheeling modes, air-propelled drifting, and quadcopter flight. Each robotic leg constitutes a total of three rotational degrees of freedom (DoF), including one DoF controlled by a brushless DC motor (after gear reduction) that drives a parallel four-bar linkage (see Figure 1B(i),(ii)), providing the major driven force for jumping, a leg servo that controls the overall rotation of the four-bar linkage (Figure 1B(iii)), and a servo that regulates the hip rotation (see Figure 1A). A TPU shock absorber is designed to avoid overextension and dampen landing impact in the jumping mode. Thrust vector control (two thrusters as a pair) is achieved via a third servo (see Figure 1C(i)). An omni-directional passive wheel is designed (Figure 1C(ii)) for heading direction change in the tricycle wheeling mode, and two passive wheels with a brake mechanism (featuring a small screw slider, see Figure 1D) provide fixed support for jumping (wheels locked) or enable the wheeling modes (wheels unlocked).

**Figure 2:**
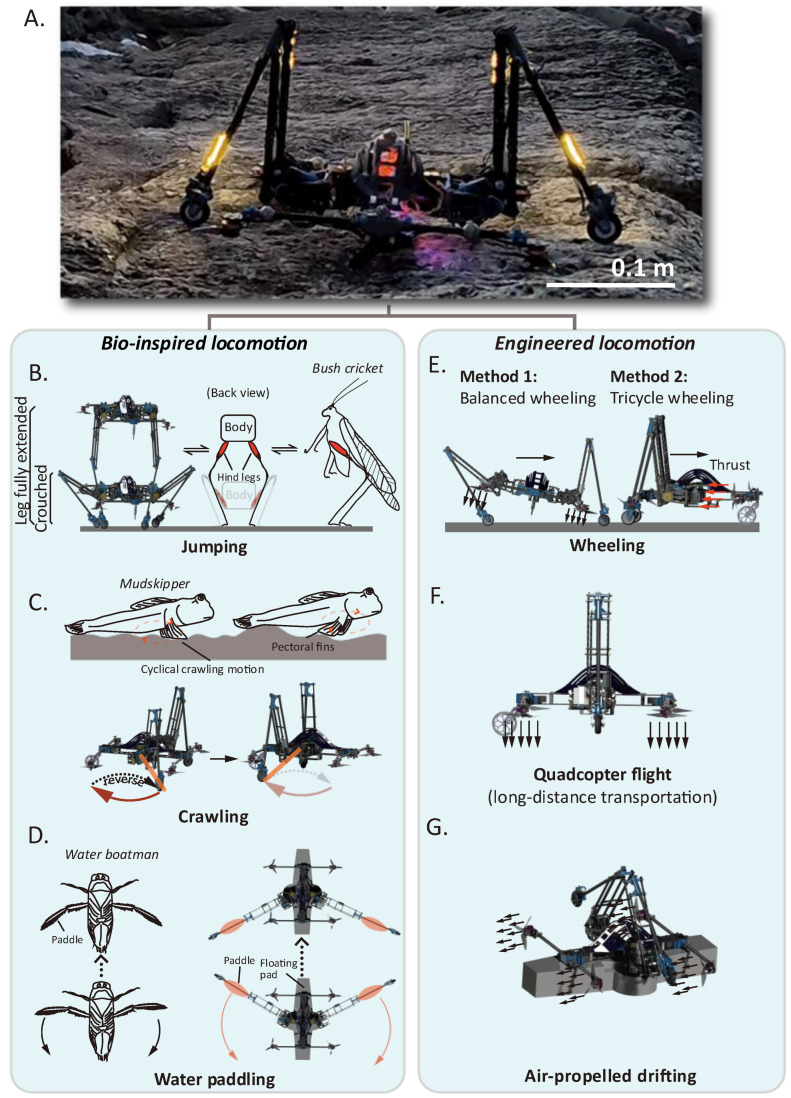
Demonstration of the proposed locomotion modes. (**A**) Image of the proposed bipedal robotic platform. (**B**) Robotic jumping strategy bioinspired by the long and thin hind legs of bush crickets. (**C**) Synchronous crawling locomotion bioinspired by the terrestrial motion of mudskippers. (**D**) Aquatic swimming bioinspired by the paddling method of the water boatmen. (**E**) Wheeling locomotion. (**F**) Quadcopter flight mode. (**G**) The air-propelled drifting locomotion in aquatic regions.

The proposed robotic locomotion modes feature a combination of bioinspired and engineered approaches, as demonstrated in Figure 2. During jumping, bush crickets (*Tettigoniidae*) leverage their long and powerful hind legs (often more than 1.5 times their body length when fully extended) to gain a long acceleration duration, enabling rapid takeoff motions for predator escapement or flight initiation [34]. Taking inspiration from this, the proposed robot also employs the synchronous extension of the two long legs, starting from a crouched pose to the leg’s full extension state (see Figure 2B). During and after the jumping acceleration procedure, the thrusters are constantly turned on, and the robot is maintained balanced using PID (Proportional-Integral-Derivative) attitude control. This method is designed to instantly overcome local obstacles and enhance robotic vertical agility (a conception proposed by Haldane et al. [35]) compared to other modes.

The crawling locomotion mode is conducted with the legs fully contracted, resembling the pectoral fins of some amphibious fish used in terrestrial locomotion mode, such as mudskippers (*Periophthalmus argentilineatus*). On land, this creature employs a cyclic motion containing a propulsive phase that retracts the pectoral fins to exert forces on the ground for forward movement, and a recovery phase that repositions the fins to the start point for the next stroke [36], as shown in Figure 2C. In the water paddling mode, an additional floating pad (made of foam) and two paddles are employed for floating and water propulsion. This locomotion pattern is inspired by the synchronous and cyclic rowing motion of the hind legs of the water boatmen (*Corixidae*), which also features a propulsive stroke and a recovery stroke [37] (see Figure 2D).

The proposed locomotion methods also include engineered solutions. The wheeling motion employs two methods: the balanced wheeling mode and the tricycle wheeling mode (see Figure 2E). The balanced wheeling method is adapted from the crouched state of the jumping mode, where the legs’ phase angle difference causes an inclination of the body and enables the generation of horizontal components of the thrust forces. During the body tilting process, the inclination attitude angle relies on the coordinated control of the legs and the PID attitude controller. The tricycle wheeling mode is controlled by the forward thrust forces (thrusters turn 90 degrees) that drive the three passive wheels. Here, the front assistive wheel is freely steerable, and the wheeling direction of the robot is controlled by thrust forces. The air-propelled drifting mode, an engineered solution designed for rapid aquatic locomotion, employs the same forward thrust source as used in the tricycle wheeling.

The quadcopter flight mode (Figure 2F) is also based on the cascaded PID attitude control, as used in jumping. Although both jumping and flying utilize aerial phases to traverse barriers, the rationale for deploying these modes varies. Flying is only used to surmount long-distance barriers where jumping is not feasible, while jumping endows higher efficiency (as legged direct propulsion wastes less energy than thrust propulsion to gain an identical height) and vertical agility in comparison to flight.

### 2.2. Analysis of the Jumping Acceleration and Aerial Phases

#### 2.2.1. Kinematics Analysis of the Jumping Process

**Figure 3:**
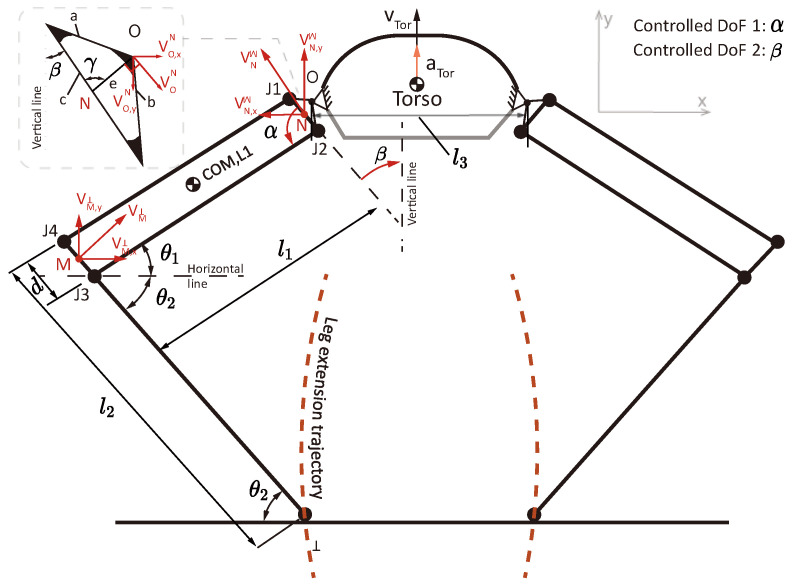
Simplified configuration of the robot in the acceleration process.

To derive the functional relationship between the angular velocity and acceleration of the leg actuators and the translational velocity and acceleration of the main body, a kinematic analysis was conducted concerning the propulsive phase of the jumping mode. Figure 3 demonstrates the simplified configuration of a random state for the acceleration process. Each leg features a four-bar mechanism, and the extension of the jumping legs is controlled by two degrees of freedom, α and β, driven by two motors. To analyze the torso vertical velocity and acceleration, an angle transformation was conducted from α and β to $\theta_{1}$ and $\theta_{2}$ for simplification of the calculation. The dimensions in Figure 3 are listed in Table 2.

Here,

(1) $$\left[\begin{array}{l} \theta_{1}\\ \theta_{2} \end{array}\right]=\left[\begin{array}{l} \pi /2-\beta \\ -\pi /2+\alpha +\beta \end{array}\right]$$

As the extension of the legs is synchronous, here we can derive the translational velocity and acceleration of the torso, $v_{tor}$ and $a_{tor}$, by analyzing only one leg. During jumping, the ground contact point T is fixed, and lower linkage can be seen as rotating around point T. The tangent velocity at point M (middle point of segment $\bar{J_{3}J_{4}}$) and its components along the x and y axes can hence be calculated:

(2) $$v_{M}^{T}=\left[\begin{array}{l} {\dot{\theta }}_{2}(l_{2}-d/2)\sin\theta_{2}\\ {\dot{\theta }}_{2}(l_{2}-d/2)\cos\theta_{2} \end{array}\right]=\left[\begin{array}{l} \left(\dot{\alpha }+\dot{\beta })(l_{2}-d/2)\sin(\alpha +\beta -\pi /2\right)\\ \left(\dot{\alpha }+\dot{\beta })(l_{2}-d/2)\cos(\alpha +\beta -\pi /2\right) \end{array}\right]$$

Assuming point M is fixed, the relative velocity of point N (the middle point of the segment $\bar{J_{1}J_{2}}$) with respect to point M is:

(3) $$v_{N}^{M}=\left[\begin{array}{l} -{\dot{\theta }}_{1}l_{1}\sin\theta_{1}\\ {\dot{\theta }}_{1}l_{1}\cos\theta_{1} \end{array}\right]=\left[\begin{array}{l} \dot{\beta }l_{1}\cos\beta \\ -\dot{\beta }l_{1}\sin\beta \end{array}\right]$$

Here, since point N conducts a circular motion over point O, the velocity of point O over N can be calculated upon a fixation assumption for point N, and:

(4) $$v_{O}^{N}=\left[\begin{array}{l} -e\dot{\beta }\cos(\gamma -\beta )\\ e\dot{\beta }\sin(\gamma -\beta ) \end{array}\right]$$

where γ=68.28° and is obtained by trigonometrical calculation. Note that here, $\dot{\beta }<0$, as the magnitude of β is decreasing during jumping. The relative velocity of point O over foot T can hence be derived by:

(5) $$v_{O}^{T}=v_{M}^{T}+v_{N}^{M}+v_{O}^{N}$$

And the vertical velocity of point O is:

(6) $$v_{O,y}^{T}=(\dot{\alpha }+\dot{\beta })(l_{2}-d/2)\cos(\alpha +\beta -\pi /2)-\dot{\beta }l_{1}\sin\beta +e\dot{\beta }\sin(\gamma -\beta )$$

Since point O is fixed to the torso, the functional relationship between the controlled DoFs α,β and the vertical acceleration of the torso can be obtained by taking the derivative of $v_{O,y}^{T}$ with time:

(7) $$\begin{array}{l} a_{O,y}^{T}=\frac{dv_{O,y}^{T}}{dt}=(\ddot{\alpha }+\ddot{\beta })(l_{2}-d/2)\sin(\alpha +\beta )+{\left(\dot{\alpha }+\dot{\beta }\right)}^{2}(l_{2}-d/2)\cos(\alpha +\beta )-\\ \left(\ddot{\beta }l_{1}\sin\beta +{\dot{\beta }}^{2}l_{1}\cos\beta )+e\ddot{\beta }\sin(\gamma -\beta )-e{\dot{\beta }}^{2}\cos(\gamma -\beta \right) \end{array}$$

#### 2.2.2. Aerial Phase Analysis

**Figure 4:**
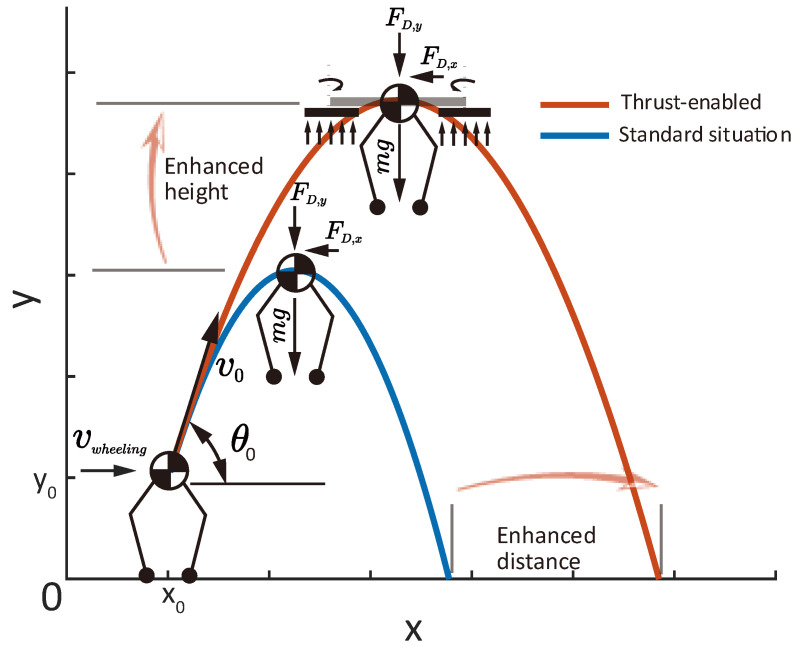
Demonstration of jumping with and without thrust force for gravity offset.

Since the robot constantly employs air thrust forces to balance and compensate for gravity during jumping, the aerial phase dynamics are different from standard parabolic motion under normal gravitational drag. Figure 4 demonstrates the comparison between the jumps with and without thrust forces to counteract gravity.

Upon takeoff, the robot attains an initial velocity $v_{0}$ and takeoff angle $\theta_{0}$ over the horizontal plane. In the vertical direction, the robot encounters gravity g, the air thrust force $\sum T_{i}$, and the air drag $F_{D,y}$. In the horizontal direction, the robot encounters the air drag $F_{D,x}$. The acceleration in the horizontal and vertical directions can be calculated by:

(8) $$a=\left[\begin{array}{l} -1/2\rho {v_{x}}^{2}C_{d,x}A_{x}/m\\ \sum T_{i}/m-g-1/2\rho {v_{y}}^{2}C_{d,y}A_{y}/m \end{array}\right]$$

where ρ is the density of the air, $C_{d,x},C_{d,y}$ are the drag coefficients in the horizontal and vertical direction, m is the total mass of the robot, and $A_{x},A_{y}$ are the reference areas in horizontal and vertical direction to establish the drag equations.

Compared to the case with a standard situation (the blue curve in Figure 4), a constant thrust force endows an effect of lowering the effective gravitational drag, i.e., $g_{effective}=g-\sum T_{i}/m$. With neglected aerial drag, the elevation of a jump can be expressed by:

(9) $$h=\frac{v_{0}^{2}\sin^{2}\theta_{0}}{2g_{effective}}+h_{takeoff}$$

Here, $h_{takeoff}$ corresponds to the robot’s standing height at the takeoff point. The introduced air thrust alleviates the gravitational drag condition, increases the duration of the aerial phase, and enhances both the leaping distance and height under the same takeoff condition $\left(v_{0},\theta_{0}\right)$.

#### 2.2.3. Motor Commands

During jumping, the actuators of the legs set the angle of α,β to generate the foot tip trajectory. In the experiments, the rotating direction of α and β are opposite. We set a mapping from α to β in order to sync the rotation of the motors and ensure a fixed foot trajectory pattern:

(10) $$\beta =\beta_{st}+\Delta \beta (\frac{\alpha -\alpha_{st}}{\alpha_{end}-\alpha_{st}})$$

Here, $\beta_{st}=45{}^{\circ},\Delta \beta =30{}^{\circ},\alpha_{st}=15{}^{\circ},\alpha_{st}=125{}^{\circ}.$

### 2.3. Synchronous Paddling-like Motion

#### 2.3.1. Crawling Method

**Figure 5:**
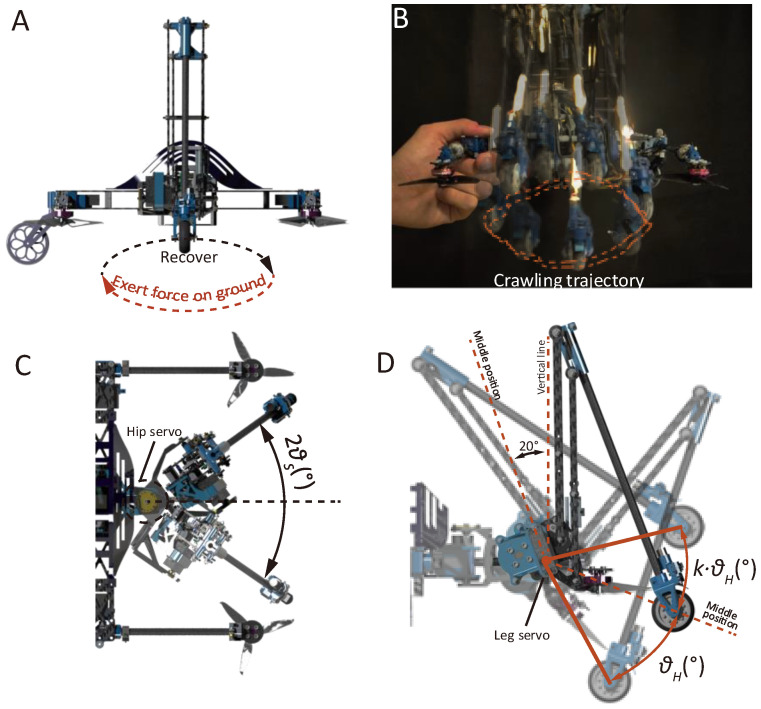
Synchronous crawling. (**A**) Demonstration of applied foot trajectory during crawling. (**B**) A video capturing the crawling trajectory. (**C**) The parameter used to control the span of the crawling trajectory. (**D**) Parameters used to control the height of the crawling trajectory.

Synchronous crawling applies the coordinated rotation of the hip and leg servos while maintaining the four-bar-mechanism leg fully contracted to generate an oval-shaped foot trajectory viewed from the side, as shown in Figure 5A,B (captured by a video analysis tool tracker). During the crawling motion, three independent parameters are set to control the pattern of the trajectory, including a span control parameter $\theta_{S}$ (Figure 5C), a paddling height control parameter $\theta_{H}$, and a ratio parameter k that independently sets the lift angle (Figure 5D). The reference position for the paddling height control corresponds to the position where the leg servo deviates 20 degrees inward from the vertical line.

**Figure 6:**
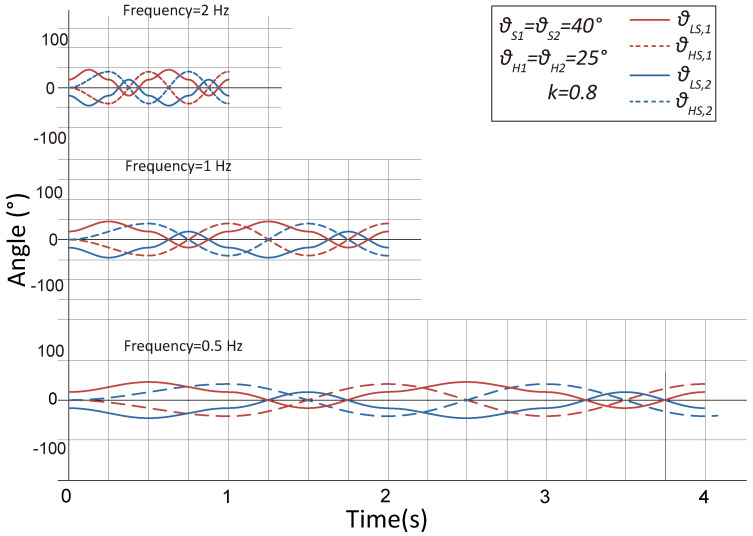
Angular commands for the crawling motion with three different frequencies.

The signals for controlling the angle of hip and leg servos of each leg, $\theta_{LS},\theta_{HS}$, are based on the same time sequence and are cyclical trigonometrical functions, as presented in the Supplementary Section S1. Figure 6 demonstrates the variation of the servo’s angular position commands over time, under three different frequencies (0.25 Hz, 1 Hz, and 2 Hz). Note that, at the beginning of the paddling motion, the swinging movement of the hip servo starts from the center line (see Figure 5C). In the field deployment of crawling, the parameters $\theta_{H},\theta_{S},$ and k can be tuned dynamically to make turns (by setting the differences of $\theta_{S1}$ and $\theta_{S2}$) or for obstacle avoidance (by changing $\theta_{H},k$ according to obstacles’ heights). In the experiments, these parameters are mapped to the sticks and rotary knobs of a radio controller (RadioLink AT9S Pro, RadioLink Electronics Co., Ltd., Shenzhen, China).

#### 2.3.2. Crawling Motion Modeling

**Figure 7:**
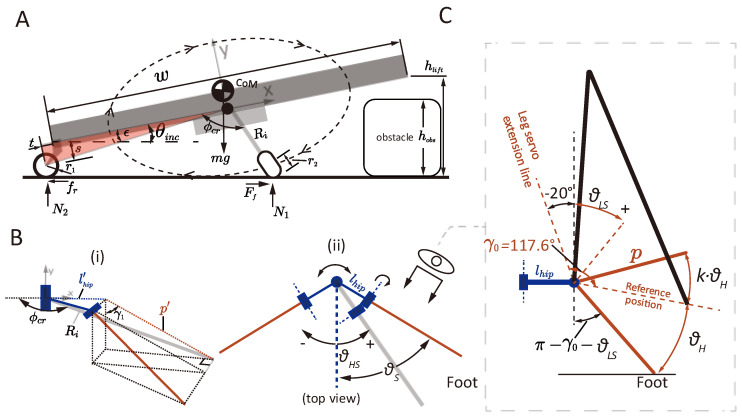
Modeling of the synchronous crawling motion. (**A**) Modeling of the crawling motion in side view. (**B**) Defining the side-view radius. (**C**) Defining parameters in the planar view of the leg unit.

During the synchronous crawling process, the robot is tail-supported by the omnidirectional wheel, and the crawling trajectory functions as a radius-tunable Wheg when viewed sidewise, as demonstrated in Figure 7A. During each crawling cycle, the trajectory lifts the head to a height $h_{lift}$ while simultaneously producing horizontal translational movement. The lateral-view radius of the crawling trajectory is defined to be $R_{i}$. The current hip and leg servos’ rotation angles are defined to be $\theta_{HS}$ and $\theta_{LS}$, respectively. The robot’s weight is mg and it applies to the robot’s center of mass. The robot encounters a pair of forces, a friction force and a normal force, at the feet ($F_{f},N_{1}$) and the tail ($F_{r},N_{2}$). To crawl over an obstacle, the robot is required to lift its head to a height $h_{lift}$ that exceeds the obstacle height $h_{obs}$. To lift the robot’s head, the leg needs to provide sufficient flipping torque that satisfies:

(11) $$2N_{1}(w/2+t+R_{\max})\geq mg(w/2+t)$$

Here, $N_{1}\approx \tau_{LS}/p$, $R_{\max}\approx p\sin\theta_{S}$, $\tau_{LS}$ is the torque produced by the leg servo, and p is the distance (a fixed value as the legs maintain full contraction) between the foot and the leg servo rotation center (see Figure 7C). $\theta_{S}$ is the defined crawling span angle (Figure 5C). By substituting $N_{1}$ in Equation (11), the torque requirement for the leg servos is obtained:

(12) $$\tau_{LS}\geq \frac{mg(w/2+t)p}{w+2t+2p\sin\theta_{S}}$$

when $\theta_{S}$ is set to be 0, i.e., the critical case that only lifts the head, the torque requirement for the leg servos reaches the maximum value, i.e.,

(13) $$\tau_{LS}\geq \frac{mg(w/2+t)p}{w+2t}=\frac{0.367\times 9.81\times (0.19/2+0.02)\times 0.083}{0.19+0.04}=0.149N\cdot m$$

The leg servos applied in the prototype have a maximum output torque of 0.31N⋅m, which satisfies the torque requirement.

To climb up an obstacle with a height of $h_{obs}$, the robot needs to lift its head to a height that exceeds the object height, i.e., $h_{lift}>h_{obs}$. Here, we first obtain the function relationship between the side-view radius of the crawling trajectory $R_{i}$ (side-view projection of foot-to-hip distance, see Figure 7B) and the servos’ joint angle by geometrical calculation:

(14) $$R_{i}=\sqrt{{l_{hip}^{\prime }}^{2}+p^{\prime 2}-2l_{hip}^{\prime }p^{\prime }\cos(\gamma_{1}+\pi /2)}$$

Supplementary Section S2.1 presents the derivation of the explicit form of $R_{i}$ regarding servo angles $\theta_{LS},\theta_{HS}$.

The head lift height can be obtained by referring to its geometrical relationship with the inclination angle $\theta_{inc}$:

(15) $$h_{lift}=(w+t)\sin\theta_{inc}+s\cos\theta_{inc}+r_{1}$$

where w = 0.19, t = 0.2, s = 0.034, and $r_{1}$ = 0.015 are the dimensions defined in Figure 7A.

The inclination angle $\theta_{inc}$ is derived by a trigonometrical relationship, and can be explicitly expressed by the servo angles $\theta_{HS}$ and $\theta_{LS}$, as presented in Supplementary Section S2.2.

The critical case for the largest head lifting height corresponds to the position where the side-view phase angle $\phi_{cr}=\pi /2$, i.e., when the side-view radius $R_{i}$ is perpendicular to the x axis of the body coordinate frame (Figure 7A), and the maximum paddling depth is reached, i.e., $\theta_{LS}=\theta_{H}-20\pi /180$. Substituting $\phi_{cr}=\pi /2$ and $\theta_{LS}=\theta_{H}-20\pi /180{}^{\circ}$ in (S8) and (S9) yields:

(16) $$R{|}_{\phi_{cr}=\pi /2}=\sqrt{p^{2}\sin^{2}(\theta_{s}+\gamma_{0}-20\pi /180)\sin^{2}\theta_{S}+p^{2}\cos^{2}(\theta_{s}+\gamma_{0}-20\pi /180)}$$

(17) $$\begin{array}{l} \theta_{inc}=\arcsin\left(\frac{\cos\varepsilon \cdot (R_{i}+r_{2})}{\sqrt{{\left(t+w/2\right)}^{2}+s^{2}+{\left(R_{i}+r_{2}\right)}^{2}+2\sqrt{{\left(t+w/2\right)}^{2}+s^{2}}\cdot (R_{i}+r_{2})\sin\varepsilon }}\right)-\varepsilon \\ \approx \arcsin(\frac{R_{i}+r_{2}}{\sqrt{{\left(t+w/2\right)}^{2}+s^{2}+{\left(R_{i}+r_{2}\right)}^{2}}})-\varepsilon \end{array}$$

**Figure 8:**
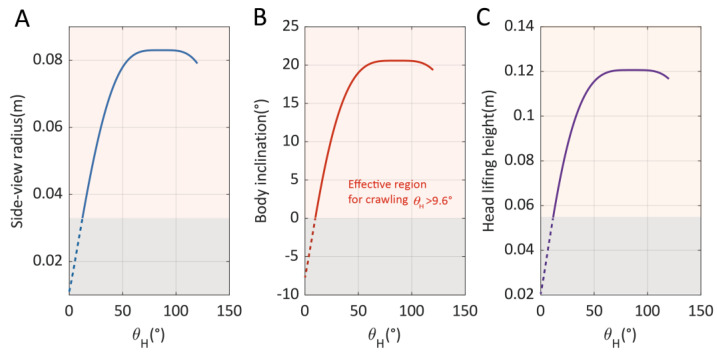
The relationship between (**A**) side-view radius, (**B**) body inclination angle, and (**C**) head lifting height and the crawling height adjusting parameter $\theta_{H}$.

According to the model, we plot the relationship between the side-view crawling radius $R_{i}$, the body inclination angle $\theta_{inc}$, and the head lifting height $h_{lift}$ and the assigned crawling parameter $\theta_{H}$, as demonstrated in Figure 8. The maximum achievable head lifting height is 0.121 m, which is reached when $\theta_{H}=74{}^{\circ}$. During crawling, the paddling height parameter $\theta_{H}$ should satisfy $\theta_{H}>9.6{}^{\circ}$, such that the maximum inclination angle exceeds zero degrees, thereby maintaining effective foot contact with ground.

#### 2.3.3. Water Paddling Design for the Swimming Mode

**Figure 9:**
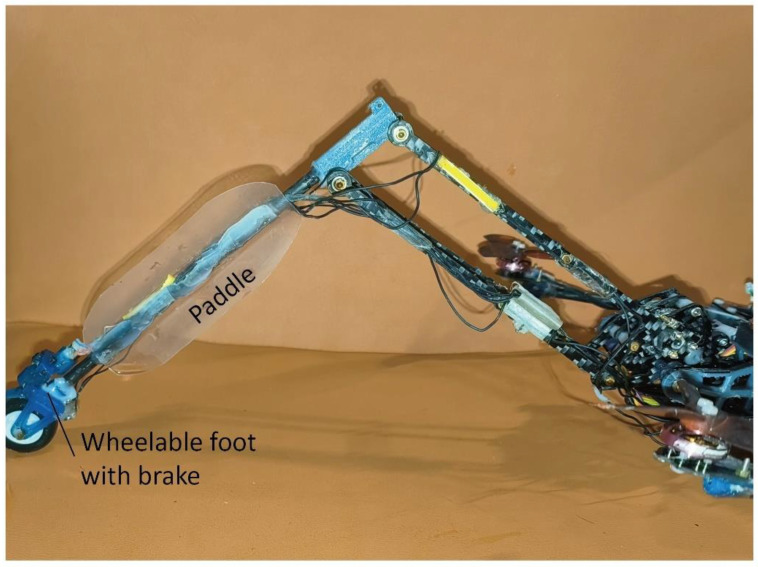
The paddle-mounted leg for the swimming mode.

The water paddling motion in swimming mode employs the same type of method as used in synchronous crawling, except that the legs are maintained extended for a broader span. A floating pad (made of foam) is used to support the robot, and two paddles are glued to the lower link with the paddling surfaces perpendicular to the tangent direction of hip rotation, as shown in Figure 9. In the experiments, we tested the influence of padding frequency on the speed and cost of transport of swimming. The corresponding reference positions (the start position of the paddling motion, as illustrated by Figure 7C) are set to ensure that the paddles are above the water surface. The settings of the leg’s angular positions are implemented in a microcontroller (Teensy 4.0).

### 2.4. Method for the Wheeling Mode

#### 2.4.1. Balanced Wheeling Mode

**Figure 10:**
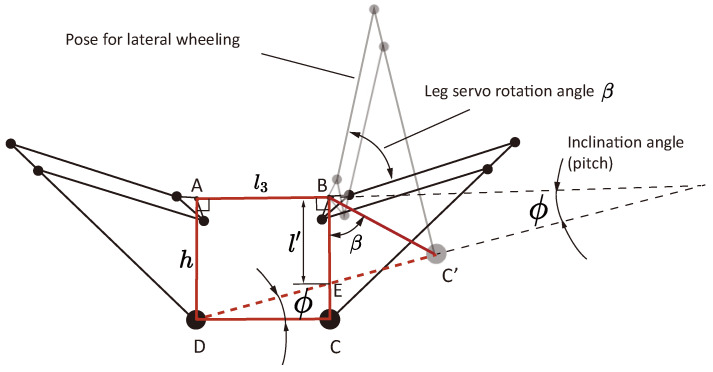
Geometrical modelling of the balanced wheeling mode.

The balanced wheeling motion is enabled by the horizontal thrust force component when an inclination of the body is created, and in this case, only the two wheels contact the ground. During balanced wheeling, to simplify the control of the leg unit, the robot only tilts one of the leg servos and maintains the other unchanged (see Figure 10). The reference position for this mode corresponds to the pose where the foot positions align with the joint centers of the leg servos. As one of the leg servos tilts an angle β, the body inclines ϕ. Note that in the tilt process, the attitude commands of the PID controller are set to tilt an identical angle.

The relationship between the leg servo tilt angle β and the inclination angle of the body ϕ can be calculated by a geometrical transform. By applying the sine rule in triangle $\Delta BEC^{\prime }$:

(18) $$\frac{l^{\prime }}{\sin(\pi -\beta -(\pi /2-\phi ))}=\frac{h}{\sin\phi }$$

An explicit form of the leg servo rotation angle with respect to a desired inclination angle can be derived:

(19) $$\beta (\phi )=\arccos(\cos\phi -\frac{l_{3}}{h}\sin\phi )+\phi$$

**Figure 11:**
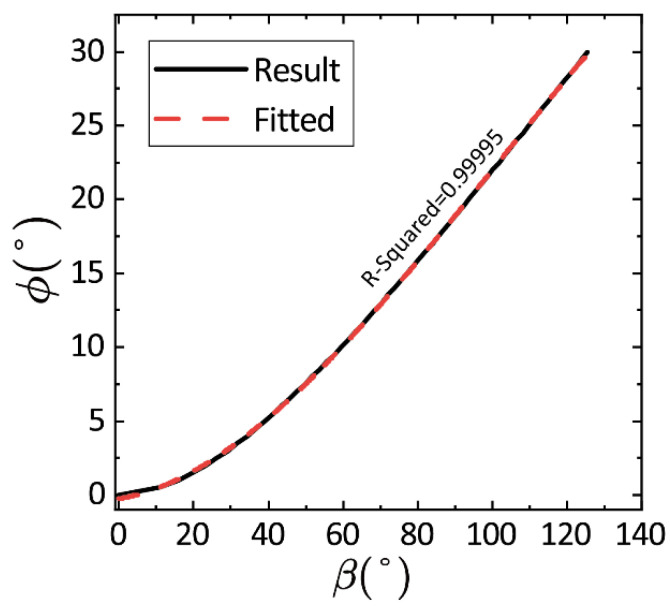
Third-order polynomial fitting for β–ϕ.

In this experiment, Formula (30) is deployed in the controller Teensy, and a prediction from β to ϕ is used for the reference setting of the attitude angle that is controlled by the thrusters, i.e., to find the expression for ϕ(β). Considering the difficulty in solving the analytical solution of the inverse of (30) for all regions of β (since multiple extrema exist), here we use polynomial fitting to find a mapping between β and ϕ in a monotonic region of ϕ, as shown in Figure 11.

The inverse relationship fitted by a third-order polynomial is hence used in the code to set the inclination command of the PID attitude controller:

(20) $$\phi =-0.25489+0.04444\beta^{3}+0.00266\beta^{2}-8.76933^{-6}\beta$$

#### 2.4.2. Tricycle Wheeling Mode

**Figure 12:**
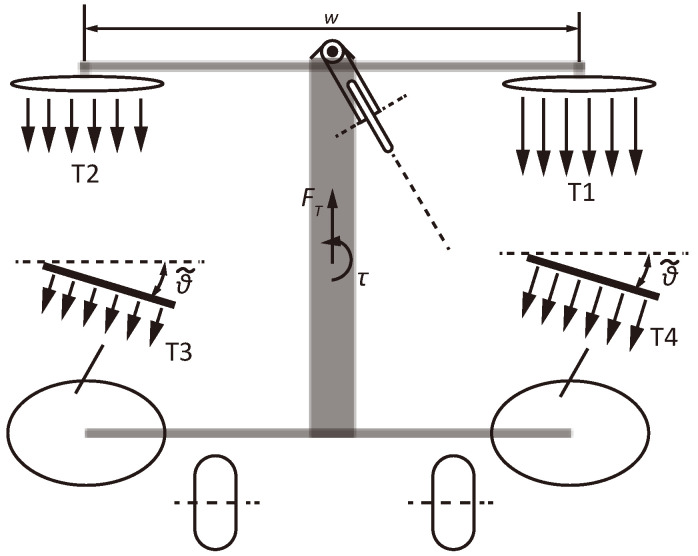
Illustration of the tricycle wheeling mode.

The tricycle wheeling mode requires the robot to conduct a pose transformation that rotates the extension plane of the two legs to be parallel, as shown in Figure 2E. In this mode, the rotation axis of the front two thrusters is perpendicular to the wheeling direction to provide the major driving force. The back thrusters tilt an angle $\tilde{\theta }$ and are employed to maintain balance (based on the same PID attitude controller used in jumping), especially when making turns. The change of wheeling direction in this mode is enabled by setting the difference in thrust force of the propellers on each side, as illustrated in Figure 12.

The torque for steering and the forward driving force, $\tau_{steer},F_{T}$, can be calculated according to the thrust force of each propeller:

(21) $$\left\{\begin{array}{l} F_{T}=T_{1}+T_{2}+(T_{3}+T_{4})\sin\tilde{\theta }\\ \tau_{steer}=(T_{1}-T_{2}+T_{4}\sin\tilde{\theta }-T_{3}\sin\tilde{\theta })\frac{w}{2} \end{array}\right.$$

Here, the thrust force is proportional to rotating speed, i.e., $T_{i}=k_{T}\omega_{i}^{2}$, and $k_{T}$ is the thrust coefficient. Then, $\tau_{steer},F_{T}$ can be expressed by the rotating speed of thrusters:

(22) $$\left\{\begin{array}{l} F_{T}=k_{T}[(\omega_{1}^{2}+\omega_{2}^{2}+(\omega_{3}^{2}+\omega_{4}^{2})\sin\tilde{\theta }]\\ \tau_{steer}=k_{T}(\omega_{1}^{2}-\omega_{2}^{2}+\omega_{4}^{2}\sin\tilde{\theta }-\omega_{3}^{2}\sin\tilde{\theta })\frac{w}{2} \end{array}\right.$$

### 2.5. PID Attitude Controller for Balancing and Flight

**Figure 13:**
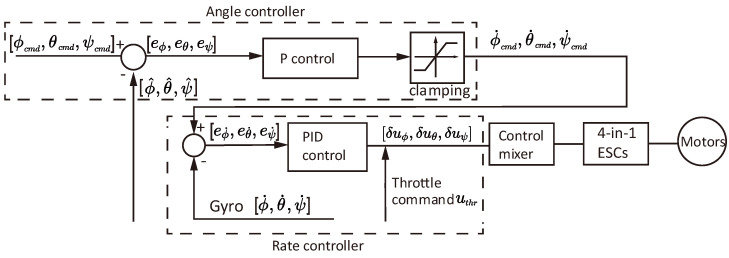
The cascaded PID controller used in the PX4 firmware [38].

The attitude control of the balancing in jumping and flight is based on the PX4 cascaded PID controller, as illustrated in Figure 13. The controller is composed of an outer angular control loop and an inner angular rate control loop.

The outer loop receives angle commands (roll, pitch, yaw: $\left[\phi_{cmd},\theta_{cmd},\psi_{cmd}\right]$) in its body frame, and inputs the error to a P controller (only for the angle loop). The P controller outputs the desired angular rate commands $\left[{\dot{\dot{\phi }}}_{cmd},{\dot{\theta }}_{cmd},{\dot{\psi }}_{cmd}\right]$ to the inner PID rate control loop, followed by a control mixer to give individual commands to the ESC (Electronic Speed Controller). The controller gains used in our prototype are listed in Table 3.

## 3. Results

### 3.1. Validation of Terrestrial Locomotion Modes

**Figure 14:**
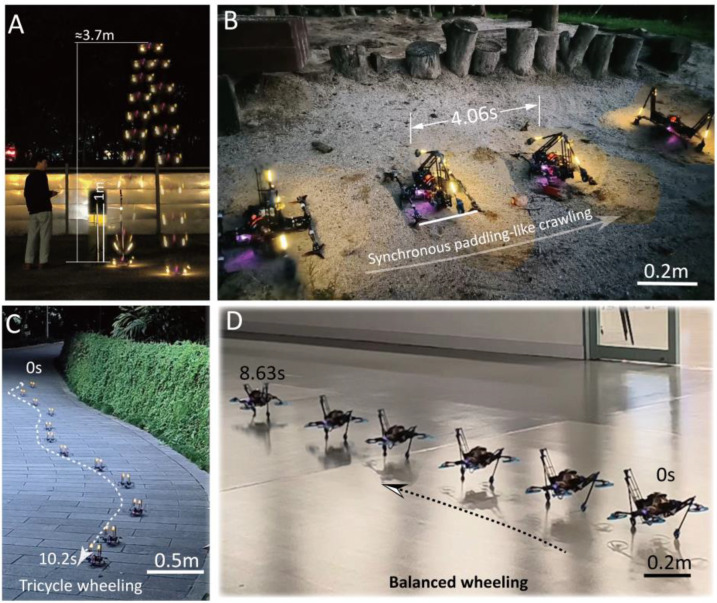
Demonstration of the terrestrial locomotion modes. (**A**) A balanced jump that reached a height of 3.7 m. (**B**) Synchronous paddling-like crawling motion on sand. (**C**) Tricycle wheeling mode. (**D**) Balanced wheeling mode.

The proposed terrestrial locomotion modes of the bipedal robotic platform, including balanced jumping, synchronous crawling, and wheeling (balanced wheeling and tricycle wheeling), were experimentally tested and validated, as demonstrated in Figure 14 and Supplementary Video S1.

In the balanced jumping mode, the robot employed air thrust force to counteract gravity, offsetting around 85% of the Earth’s gravitational force. This enables the 370 g bipedal robot to reach an impressive height of 3.7 m (see Figure 14A), a record higher than most jumping machines in the literature. During this experiment, the throttle command of the robot was controlled at a constant value before and after the jump. Upon landing, since a great proportion of gravity was compensated, the robot landed with a relatively lower velocity compared to the case of jumping without gravity offset, though achieving an identical height.

The synchronous crawling mode was experimentally evaluated on a sand substrate (Figure 14B). During the trial, the robot maintained a paddling frequency of 0.5 Hz, achieving an estimated forward velocity of 0.1 m/s. Empirical observations demonstrated that reduced actuation frequencies improved foot-substrate contact stability. Conversely, higher frequencies resulted in pronounced foot slippage (especially up to 2 Hz), attributed to incomplete force transmission during the shortened contact phase.

The tricycle wheeling was tested on an inclined and winding road, and it achieved a notably higher speed compared to the crawling motion (see Figure 14C). The steering of the robot in this wheeling mode was manually controlled using a radio controller. Figure 14D demonstrates the movement that employs the propellers’ horizontal thrust force component for lateral wheeling, using the method presented in Section 2.4.1. This mode revealed a lower speed compared to the tricycle mode, as only part of the thrust force is effective in driving the wheeling motion.

### 3.2. Synchronous Crawling on Various Terrains

**Figure 15:**
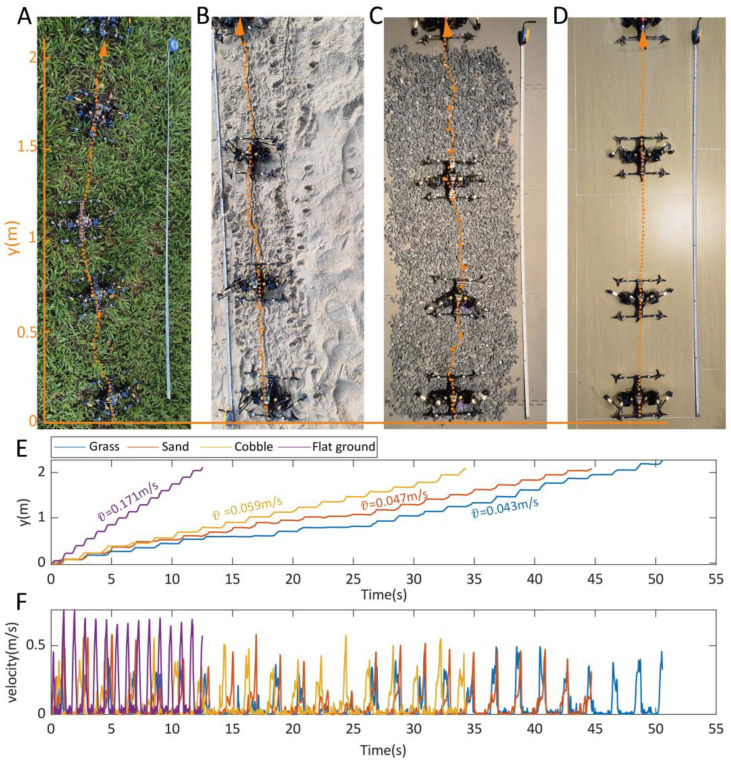
Testing of the synchronous crawling locomotion on different terrains. (**A**) Locomotion on a medium-density grassland. (**B**) Locomotion on soft sand. (**C**) Locomotion on cobbles. (**D**) Locomotion on flat ground. (**E**) The captured displacements versus time. (**F**) Locomotion velocity versus time in the tests. The arrows and the dashed lines refer to the motion directions and trajectories, respectively.

To evaluate the adaptiveness of synchronous crawling locomotion to uneven terrain, the robot was experimentally tested on four distinct substrates: medium-density grassland, soft sand, cobblestones, and flat ground (control condition), as demonstrated in Figure 15 and Supplementary Video S2. The locomotion on these terrains was manually controlled using a radio controller, by employing the method presented in Section 2.3.1. On the flat ground, the robot achieved the highest crawling average speed of 0.164 m/s (around 86% body lengths/s). Without interference of uneven terrain features, locomotion on flat ground features a quasi-straight line, as revealed in Figure 15D, whereas the test on other uneven terrains saw adjustment of head direction and trajectories (Figure 15A–C). The trajectories of all groups were captured by a video analysis tool tracker. According to the captured displacements and velocity (Figure 15E,F), the crawling motion features incremental movements, and the robot’s velocity was cyclical. The peak crawling speed the robot can reach is 0.76 m/s (about four times its body length/s). The paddling frequency in Figure 15A–C was set to 0.5 Hz, with $\theta_{H}=25{}^{\circ},\theta_{S}=40{}^{\circ},k=0.8$. For the uneven terrains, higher frequencies (e.g., 1 Hz) would pose apparent slippage and ineffective paddling motion. To test the maximum flat-ground speed, we increased the crawling frequency to 1 Hz and maintained the rest of the parameters unchanged. The average cost of transport (COT) of crawling on flat ground attains a value of 5.11, as detailed in the calculation in Supplementary Section S3.

**Figure 16:**
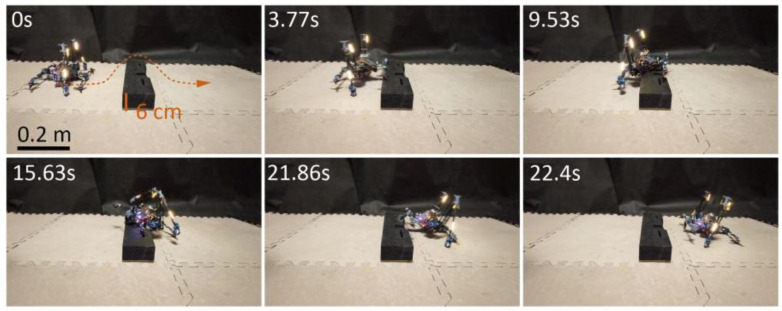
Crawling over a 6 cm-high object by adaptively changing paddling depth. The arrow and the dashed line refer to the motion direction and trajectory, respectively.

By increasing the paddling depth control parameter $\theta_{H}$ (discussed in Section 2.3.1) from the flat terrain setting ($\theta_{H}=25{}^{\circ}$) to a climbing setting ($\theta_{H}=50{}^{\circ}$), the robot demonstrated the ability to crawl over a 6 cm-high obstacle, within a duration of 22.4 s, as shown in Figure 16 and Supplementary Video S2.

### 3.3. Object Transfer Tasks

**Figure 17:**
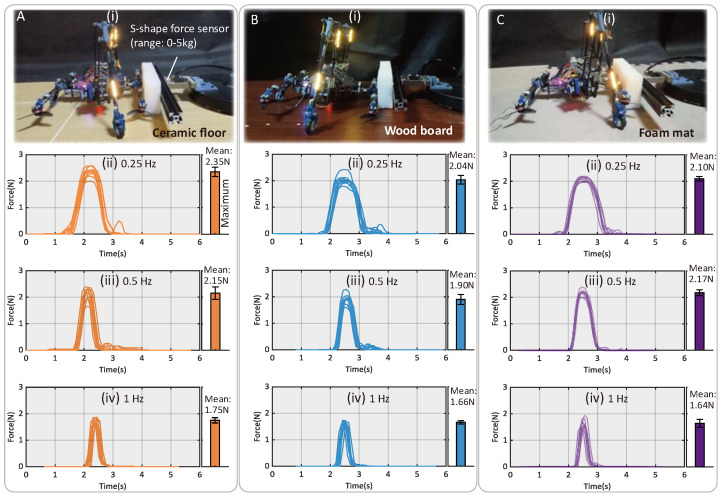
Test of push force with three different paddling frequencies on three types of substrates. (**A**) Push force test on ceramic floor. (**B**) Push force test on a varnished wood board. (**C**) Push force test on foam pad.

Our robot demonstrates the ability to conduct object transfer tasks by leveraging the pushing force resulting from the synchronous crawling motion. The push force of the robot under different substrates (ceramic floor, wood board, and foam mat) was statistically measured using an S-shaped force sensor (measurement range 0–5 kg), as demonstrated in Figure 17.

We conducted ten tests for each group. And the results also reveal that the frequency of leg movement can influence the magnitude and duration of the push force profile. The maximum push force was observed in the group tested on the ceramic floor at a paddling frequency of 0.25 Hz, reaching a mean value of 2.35 N (65% of the robot’s weight). Overall, for all groups, the increase in paddling frequency reduced both the duration and maximum value of the force applied. At 1 Hz, the maximum forces were under 2 N for all substrates. The error bars in Figure 17 correspond to the standard deviation of the ten trials.

**Figure 18:**
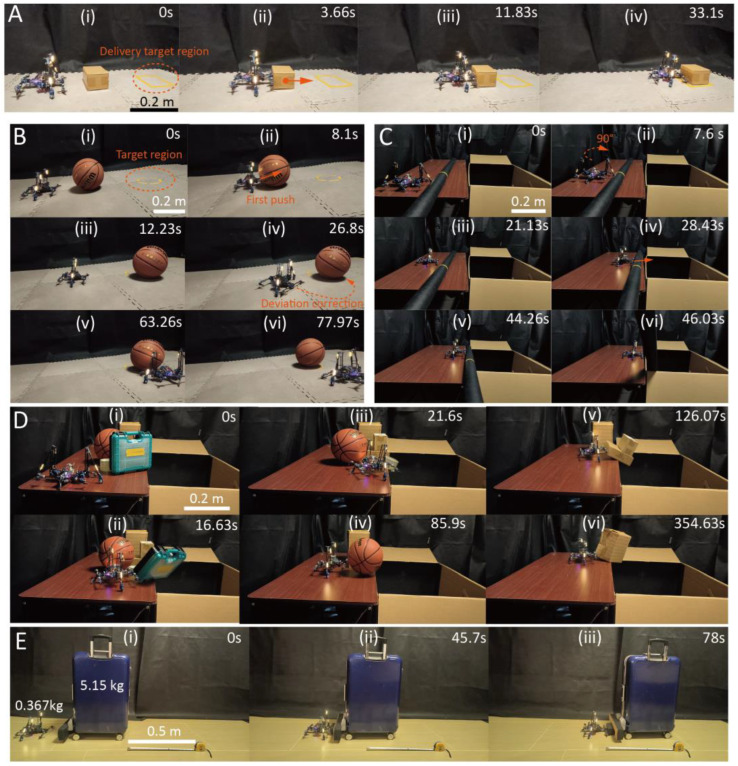
Object transfer tasks. (**A**) Transferring a 0.2 kg parcel to a target area. The arrow corresponds to the motion direction. (**B**) Moving a basketball to a desired circular region. (**C**) Pushing a roll of cloth of 1.06 kg. (**D**) Transferring multiple objects into a storage box. (**E**) Push a luggage that is 14 times its weight for 0.5 m.

According to the trend observed in the statistical tests, we deployed the robot in a series of object transfer tasks with a paddling frequency of 0.25 Hz. As illustrated in Figure 18A and Supplementary Video S3, the robot executed a parcel transfer operation to a designated target area, completing the task within 33.1 s while ensuring precise positioning of the parcel within the target zone through fine-tuned positional adjustments. To showcase the controllability of this function, a more complex task was designed, requiring the robot to transfer a basketball into a confined circular region. Due to the inherent mobility of spherical objects, which are prone to uncontrolled displacement, this task necessitated meticulous regulation of incremental pushing motions. As shown in Figure 18B, the robot successfully addressed this challenge, achieving targeted placement of the basketball within the circular boundary in 77.97 s.

Figure 18C,D exemplifies the robotic system’s capacity for object transfer into a confined storage box. In Figure 18C, the robot successfully transported a 1.06 kg cloth roll to the designated box, completing the task in 46 s. Figure 18D presents a more complex multi-object transfer task, wherein the robot sequentially transferred six items with different masses and shapes, including a toolbox (0.70 kg), adhesive tape (0.124 kg), a basketball (0.618 kg), and three parcels (0.201 kg, 0.261 kg, and 0.62 kg), into the storage box without intermediate repositioning. In this sense, the robot was not only required to conduct motion on the narrow table but also to find suitable positions to push the object.

In the final task, the robot was challenged to move a wheeled luggage that is 14 times its weight. Figure 18E (and Video S3) demonstrates the robot moving the luggage for 0.5 m, with a duration of 78 s.

### 3.4. Locomotion in Aquatic Regions

**Figure 19:**
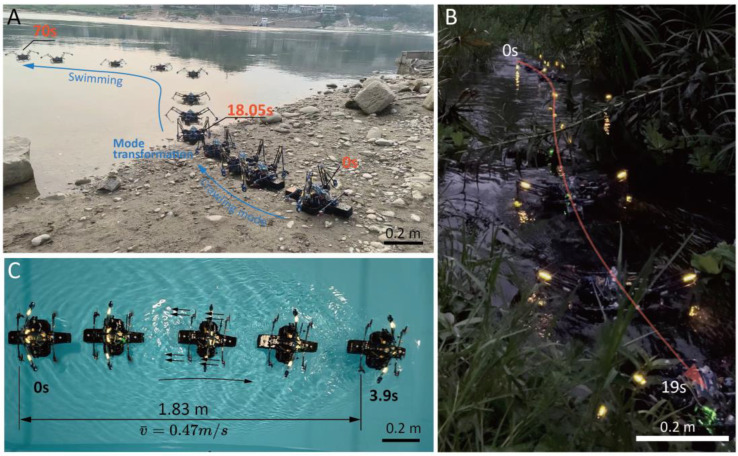
Demonstration of the aquatic locomotion modes. (**A**) Locomotion mode transformation from synchronous crawling to aquatic paddling in a river. (**B**) Synchronous paddling-like swimming in a narrow stream. (**C**) The air-propelled drifting mode.

We tested the water-paddling swimming locomotion mode in outdoor aquatic environments, including an open river and a narrow stream (Figure 19A,B and Video S4). On the riverbank, the robot employed a synchronous crawling method to navigate the gravel terrain by elevating its body. Upon entering the water, the robot extended its legs to a new reference position (introduced in Section 2.3.2) for paddling. The adjustments to paddling span and direction during swimming utilized the same method as in crawling mode, ensuring consistent motion control across both terrestrial and aquatic phases, while the elongated span helps to produce sufficient propulsion force from water. The paddling frequency in water was set to be 1 Hz.

In the swimming tasks on the narrow stream (Figure 19B), the robot needed to adjust its heading direction and make instant paddling motions to avoid crushing onto the bank or being entangled by the grass. In this stage, the robot was manually controlled using a radio controller.

The air-propelled drifting mode employs the same method as the tricycle wheeling, using forward thrust force as the primary driving force. Compared to paddling-based swimming, this mode showcases a higher locomotion velocity. Figure 19C demonstrates this swimming mode in static water with throttle command of a mere 15% (in a constant-speed phase). The robot initiated its motion from rest and reached a distance of 1.83 m in 3.9 s, reaching an average velocity of 0.47 m/s. With the throttle commands exceeding 30%, the robot is expected to gain much higher driving forces. However, as the thrust forces point forward, the hydraulic drag force acts on the floating pad backward. The two forces form a moment that causes the robot to incline forward. In critical cases, the front two propellers were observed to contact the water surface. This phenomenon can significantly interfere with the intended motion direction and pose difficulty for control.

### 3.5. Aquatic Swimming Statistical Tests

We conducted statistical tests to analyze the locomotion performance (speed and energy efficiency) of the paddling-based swimming and the air-propelled drifting modes. For the paddling-based swimming, the relationship between paddling frequency, paddle length, and swimming speed and energy efficiency was analyzed. The speed and energy efficiency of the air-propelled drifting mode were analyzed under a variety of throttle commands.

#### 3.5.1. Speed, Power, and Cost of Transport (COT) Influenced by Paddling Frequency

**Figure 20:**
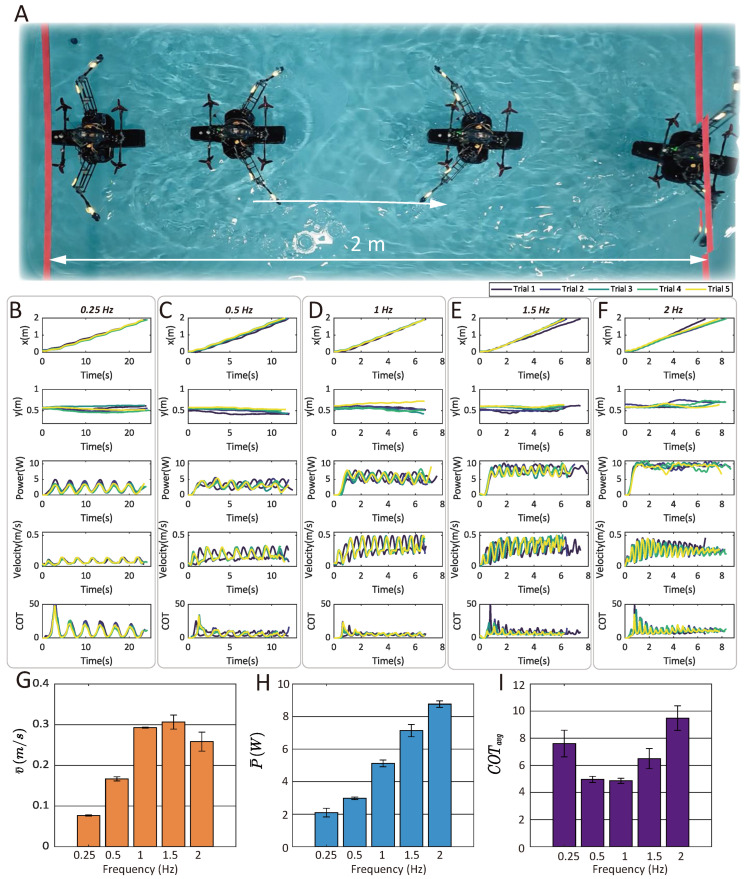
Statistical tests on the water-paddling-based swimming under different frequencies. (**A**) The image of the paddling-based swimming. The arrow refers to the motion direction. (**B**–**F**) The recorded trajectory, speed, power, and instantaneous COT with five different paddling frequencies (from 0.25 Hz to 2 Hz). (**G**–**I**) The influence of the paddling frequency on the average swimming velocity, power consumption, and energy efficiency (denoted by average COT).

Figure 20 demonstrates the water paddling locomotion and the corresponding trajectories, velocity, power, and instantaneous cost of transport (COT=P/mgv) under five different groups of paddling frequency (from 0.25 Hz to 2 Hz). Five trials were conducted for each experimental group. The results indicate that the optimal paddling frequency occurs at 1 Hz. Within this group, the robot attained the lowest average cost of transport among the five trials and achieved an average velocity of 0.293 m/s (mean of five trials), while a paddling frequency of 1.5 Hz resulted in the highest average swimming speed (0.307 m/s). Figure 20G–I illustrates the direct relationship between the paddling frequency and the three investigated variables (i.e., ${\bar{v}}_{avg}$), presented as mean ± SD. Each reported mean corresponds to the average of five independent trial means. The mean values for each individual trial are presented in Table 4. Note that the average COT in Table 4 is defined as $\bar{P}/mg\bar{v}$ and is different from the average of instantaneous COT ($\bar{P/mgv}$). Overall, a positive correlation exists between the power consumption and the paddling frequency. The results reveal that increasing the paddling frequency from 0.25 Hz to 1 Hz resulted in a reduction of COT (an increase of energy efficiency). However, frequencies exceeding 1 Hz (1.5 Hz and 2 Hz) exhibited no further obvious enhancement in locomotion speed. Instead, these higher frequencies demanded greater power input, leading to a suboptimal increase in COT compared to the 1 Hz condition. The trajectories and velocity were video recorded and captured using a video analysis software (Tracker: version 6.1.7). The power consumption was measured using a voltage and a current sensor and sent back to the computer via a wireless module (HC-12, Guangzhou Huicheng Information Technology Co., Ltd., Guangzhou, China).

#### 3.5.2. Speed, Power, and Cost of Transport (COT) Influenced by Paddling Span

**Figure 21:**
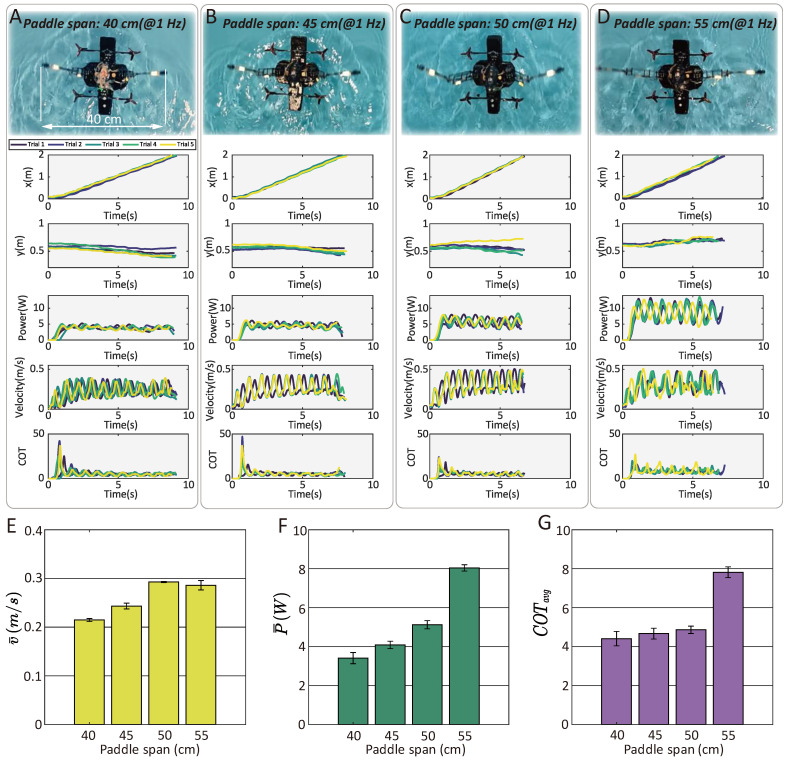
Statistical tests on the paddling-based swimming with different paddle spans. (**A**–**D**) The swimming trajectory, speed, power consumption and instantaneous COT with four different paddle spans (from 40 cm to 55 cm). (**E**–**G**) The influence of paddle span on the average locomotion velocity, power consumption and energy efficiency (denoted by average COT).

In addition to paddling frequency, our results reveal that the locomotion speed and energy efficiency of paddling-based swimming are also influenced by the leg extension ratio (i.e., the paddle span). To investigate this relationship, we conducted an experimental study using four paddle spans (40 cm, 45 cm, 50 cm, and 55 cm) in the same static water environment shown in Figure 20. The paddling frequency was fixed at 1 Hz as a control variable, and five trials were performed for each group to assess average performance. Figure 21 illustrates the recorded swimming trajectories (represented by their *x*- and *y*-coordinates), velocity profiles, real-time power consumption, and cost of transport (COT).

The results indicate a positive correlation between paddle span and swimming speed in all groups with paddle span lower than 55 cm. The maximum average velocity (0.293 m/s) is achieved at a 50 cm paddle span (consistent with the configuration in Figure 20D). The intuitive relationship between the paddle span and the averaged speed, power, and average cost of transport are presented in Figure 21E–G (mean ± SD). As the paddle span increases to 55 cm, the average power consumption notably increases, from an average of 5.12 W observed in the group with a 50 cm span to a value of 8.04 W. The average velocity in the last group, however, did not show notable improvement (0.286 m/s). This results in a reduced horizontal locomotion energy efficiency. The average cost of transport reaches an average minimum value of 4.41 with the smallest span (40 cm), while increasing to 7.82 with span = 55 cm. The comprehensive statistical data for individual trials (average values) are presented in Table 5.

#### 3.5.3. Correlation Between the Air-Propelled Drifting Speed, Power, and COT and Thrust Command

**Figure 22:**
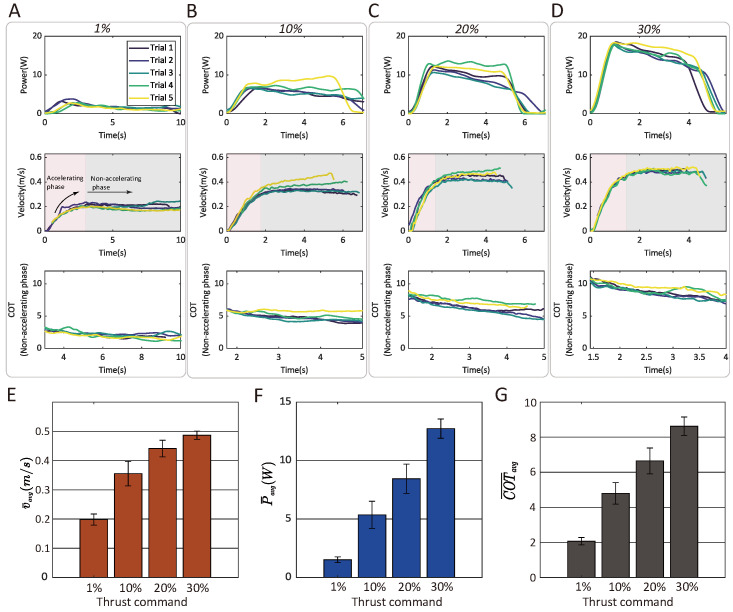
Statistical analysis of the air-propelled drifting mode under varying thrust commands. (**A**–**D**) Aquatic drifting velocity, power consumption, and instantaneous COT under four groups of thrust commands (1%, 10%, 20%, and 30%). (**E**–**G**) The influence of the level of thrust command on average swimming velocity, power, and energy efficiency (denoted by average COT in the non-accelerating phase).

In air-propelled drifting mode, the drifting speed is directly controlled by the thrust command. To evaluate locomotion performance under varying inputs, systematic tests were conducted using four thrust command levels: 1% (slightly above the PX4 flight controller’s arming threshold), 10%, 20%, and 30%. Each command group underwent five trials in a controlled environment, with only the front two thrusters activated, as they provided sufficient propulsion for stable drifting (Video S4). The resulting power, speed profiles, and efficiency metrics (COT) are illustrated in Figure 22.

The peak drifting speed was observed with the largest command of 30%, attaining an average value of 0.4866 m/s (five trials averaged, see Figure 22E). Note here that the average velocity in Figure 22E considers the acceleration process. The COT plot presents the non-accelerating phase of the motion (the time when the velocity exceeds its average value) for intuitive comparison among the four groups. Inverse to the trend of the velocity, the energy efficiency exhibited a negative correlation to the commands (see Figure 22G). The most energy-efficient setting was observed in the group with a 1% command, reaching a minimal average COT of 2.07. Efficiency declined progressively at higher throttle levels, with increased propeller speeds correlating to greater non-propulsive energy losses. Specifically, this can be attributed to the larger fraction of input power dissipated as acoustic noise, mechanical vibrations, and water-wave generation rather than contributing to translational motion. Comprehensive performance data, including individual trial averages, are summarized in Table 6 for reference.

### 3.6. Jumping Assistive Flight

**Figure 23:**
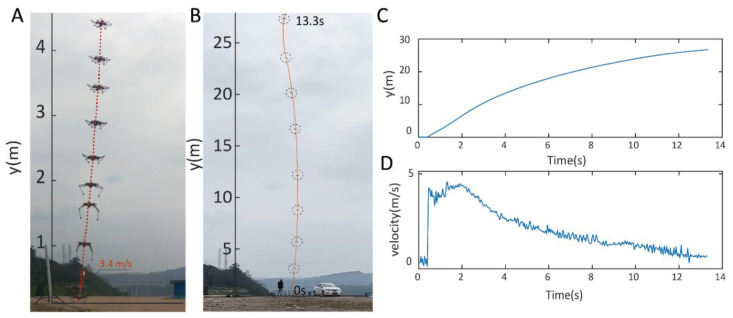
Jumping-assisted flight. (**A**) Demonstration of motions of the robot and the jumping legs in the initial stage of flight. (**B**) Complete motion of the jumping assistive flight. (**C**) Elevation–time plot. (**D**) Velocity–time plot.

The quadcopter flight mode is specifically designed to enable long-distance transitions in scenarios where other locomotion methods are impractical. In this mode, the jumping legs serve as an assistive mechanism to rapidly initiate flight, a strategy inspired by natural examples such as birds and insects (e.g., bush crickets). To evaluate this motion pattern, we tested the robot in a vertical jumping flight task, measuring its elevation and speed. Figure 23A illustrates the robot’s trajectory during the first four meters of its jumping-assisted flight. The propulsion generated by the jumping legs allowed the robot to achieve an instantaneous takeoff velocity of 3.4 m/s. Once airborne, the robot contracted its legs to maintain a crouched pose. Compared to direct thrust propulsion, which requires continuous vertical acceleration, the jumping assisted method only requires the robot to maintain thrust force near a hovering level, enabling sustained flight and ascent to a height of 26.7 m (Figure 23B,C) over 13.3 s. Figure 23D further details the velocity profile throughout the jumping-assisted flight process (captured by a video analysis tool, Tracker).

The average power consumption in the jumping assistive flight is 292 W. The system consumes a total energy of 3.951 KJ to achieve the apex of 26.7 m, based on an estimation method of measuring the energy discharged to the battery voltage level when the robot is at the apex, similar to the way used in [39] (see the measurement method in Supplementary Section S4). In this case, the average COT for vertical ascent (E/mgh) is 41.1.

## 4. Discussion

In this work, we presented a multi-terrain bipedal robotic platform that can adapt its locomotion policies to traverse terrains across land, water, and air. The robot features seven locomotion modes, including (1) jumping, (2) synchronous crawling, (3) tricycle wheeling, (4) balanced wheeling, (5) water-paddling-based swimming, (6) air-propelled drifting, and (7) flight. The locomotion modes were validated through field experiments or indoor statistical tests. In the balanced jumping mode, owing to the thrust compensation for gravity, the conducted jumping test reveals the superiority of the robot in attaining a jumping height (3.7 m). This may endow the robot with great potential to conquer large spatial obstacles in real environments, such as large debris in disasters. On uneven terrains, the robot can leverage its crawling mode for meticulous position and heading direction adjustment. This mode also endows the robot with the capability to transfer objects through the redundant push forces from the crawling motion, which unlocks parcel delivery tasks in target regions. The push force of this mode was statistically studied, and an optimal crawling frequency at 0.25 Hz can create a push force that is 65% of the robot’s weight, enabling the robot to transport an object 14 times its weight (a wheelable luggage). In regular flat terrains, the robot can transition to the wheeling mode for quick and efficient motions. Traversing beyond terrestrial environments, the robot demonstrated to conduct aquatic swimming locomotion in a wild river and stream and leveraged the jumping legs to initiate flight maneuvers.

Owing to the integrative design and mechanism reuse, our system minimizes the use of additional components for multi-functionality. For example, the two major structures, the leg unit and the flight unit, are reused in multiple locomotion scenarios:

- **Leg unit**: crawling, jumping, paddling, and balanced wheeling (four modes).
- **Flight unit**: jumping, flight, drifting, and tricycle/balanced wheeling (five modes).

In terms of structural reuse rate, the proposed platform vies with state-of-the-art multimodal systems. For example, the Caltech M4 [26] achieved a maximum reuse rate of four (via its wheel and leg mechanisms), and the multifunctional bipedal robot Leonardo [39] attained the same rate with its flight unit. A qualitative comparison to some existing platforms is presented in Table 7, demonstrating the proposed system’s advantages in mechanism functional integration.

**Figure 24:**
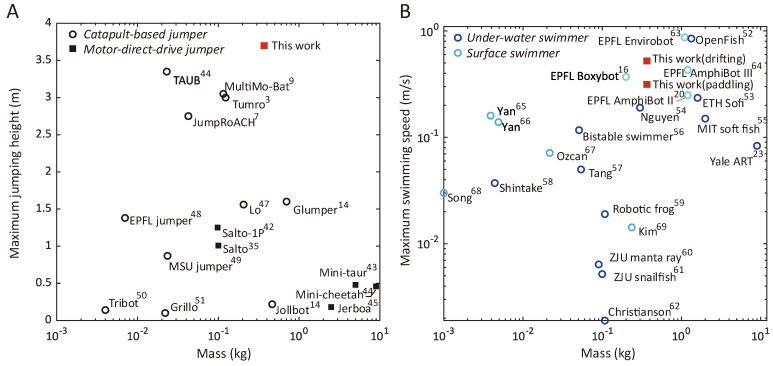
Comparison of locomotion performance regarding (**A**) jumping and (**B**) swimming to some existing specialized robotic platforms.

In field robotics, the pursuit of multifunctionality often compromises individual performance through trade-offs such as added weight, increased energy consumption, or conflicting workspace. For example, researchers from Caltech developed a bipedal aerial-terrestrial platform, “Leonardo” [39], that employs air-assisted walking as one of the primary locomotion modes. Yet, on flat ground, this mode runs at a modest speed (0.2 m/s) and consumes a notable power (544 W), resulting in a low energy efficiency (COT = 108). In comparison, the proposed system in legged crawling mode operates at a comparable speed, but consumes a power of a mere 3.1 W, attaining an average COT of 5.1, 21 times smaller than Leo’s. Crucially, this efficiency does not come at the expense of other functionalities. The robot’s jumping performance surpasses most specialized platforms in the literature [3,7,9,14,35,42,43,44,45,46,47,48,49,50,51]. To the best of our knowledge, its current leap record is higher than all existing robots using the motor-direct-drive method (the previous highest record was achieved by Salto-1P [42], at 1.25 m), and vies with vast catapult-based jumpers in the literature (see a comparison in Figure 24A). The gravity offset strategy may endow potential for achieving higher leap records via optimizing its leap mechanism energy output or compensating for gravity more with increased thrust command. Additionally, the assistance from the thrust results in a reduced effective gravity, meaning less prone to damage upon landing. In aquatic mode, the robot achieved a maximum average speed of 0.51 (1.71 body lengths/s) m/s in drifting and 0.32 m/s in paddling, which are comparable to some existing underwater [23,52,53,54,55,56,57,58,59,60,61,62] and water-surface [16,20,63,64,65,66,67,68,69] swimmers (see Figure 24B). The proposed drifting and paddling swimming modes outperform most examples.

Despite the advantages in locomotion performance and the versatility features, the current design still encounters issues arising from the conflicts of working spaces in different modes, particularly from the use of the floating component in the aquatic modes. For the other modes, the structure causes around 10% of mass increase and introduces additional drag, which could hinder the performance of modes with fast translational velocity, such as wheeling and jumping. On the other hand, due to the size of the floating pad, the leg’s working space is partly confined, and the space for the assistance wheel for the tricycle wheeling is occupied. To address these limitations, future iterations will prioritize a compact redesign of the floating foam, optimizing its geometry to minimize mass and drag while ensuring compatibility with the leg’s workspace and auxiliary components.

The integration of the proposed multimodal locomotion system endows the robot with versatile application potential across diverse scenarios. In search-and-rescue operations, the robot’s crawling mode enables navigation of uneven, unstructured terrain, while its jumping capability facilitates traversal of large obstacles (e.g., collapsed debris), complemented by energy-efficient wheeling for rapid mobility on urban paved surfaces. For environmental monitoring, the hybrid aquatic and aerial locomotion capabilities allow the robot to collect water samples from inaccessible aquatic environments via swimming and return via flight for expedited analysis, which is a critical advantage in pollution tracking or ecological surveys. Furthermore, when equipped with integrated sensors and imaging systems, the platform could be deployed in remote wilderness areas to observe wildlife colonies with minimal human disturbance, mitigating risks to researchers while reducing behavioral intimidation of sensitive species. These multifunctional capabilities position the robot as a transformative tool for applications requiring adaptability across land, water, and air.

Despite the presented locomotion versatility, the robot can encounter challenges in real-world environments. For example, the thrust-assisted jumping method could be strongly influenced by external wind disturbances in extreme weather, as the attitude is controlled to be horizontal in the aerial phase, which leads to an unpredictable landing point. In the swimming mode, the current prototype can only work in static or relatively slow aquatic regions. Extreme cases, such as fast flows or waves, can significantly interfere with the paddling or drifting motions, causing loss of heading direction control or even flipping the body backward. To better adapt to the discussed environmental challenges, the robot demands further improvements in its design and locomotion control methodologies. In the jumping mode, it first needs to be equipped with directional jumping capabilities. To counteract the discussed wind disturbance, one feasible solution is to take in aerial attitude control and use the horizontal thrust force component to compensate for the encountered positional errors while tracking a desired parabolic leap trajectory. In the swimming mode, to prevent heading direction change in fast and unpredictable hydraulic flows, we aim to develop a yaw angle stabilizer in our future work. This can be realized by introducing feedback adjustment in the difference of two paddle span angles, i.e., using the PID method to control $\Delta \theta_{S}=\theta_{S1}-\theta_{S2}$. Another feasible solution could be using the PID method to adjust the thrust force of the propellers as they tilt forward.

**Figure 25:**
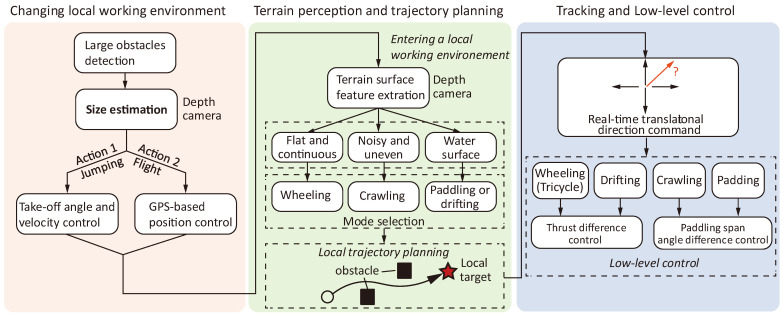
A simplified workflow for autonomous operation.

At the current stage, the proposed research focuses on the individual locomotion patterns. The control of each locomotion mode is primarily based on manual operation via a remote radio controller. To transition to autonomous operation, a comprehensive framework for perception, policy-making, trajectory planning, and lower-level control needs to be established. For example, one viable operation workflow could follow the pattern presented in Figure 25. In this framework, the goal of the robot is to reach a designated global location specified via GPS. The task can be separated into two general phases: (1) the change of local working environment and (2) locomotion within a local working environment. Here, the local working environment refers to a small patch of a region where crawling, wheeling, or swimming is feasible. At a random location, the system first needs to develop a vision-based algorithm to decide whether it needs to change the local working environment, by detecting large obstacles and estimating the sizes. The obstacle estimation guides the policy making of jumping (e.g., if under 3 m, then adjust the robot’s take-off angle and velocity to overcome the obstacle) or flight (via GPS-based position control). Upon entering a new local working environment, the robot performs terrain surface feature extraction (possibly also based on a vision-based method), and takes the selection of locomotion policies (e.g., wheeling locomotion for flat and continuous surface, crawling for noisy and uneven surface, and transforming into paddling or drifting if terrain surface features dynamically change, i.e., water surface). Once a locomotion policy is deployed, a trajectory planning module (potentially a learning-based algorithm, by fusing depth camera and GPS data as input) generates a viable path to navigate around local small obstacles, providing real-time heading direction command to the low-level control module. The low-level control module then manages the linkage movement (for crawling and water paddling) or thrust modulation (for wheeling or drifting) using a PID-based method.

## Figures and Tables

**Table 1:** The robotic functions and the corresponding methods and dedicated scenarios.

Environment	Functions	Method	Dedicated Scenarios
Terrestrial	Jumping	Leg synchronous extension while balanced by air thrust force	Overcoming large local obstacles
	Crawling	Synchronous paddling-like pulling motion	Rough terrain (e.g., cobbles, sand, and grassland)
	Balanced wheeling	Balanced while using horizontal thrust force to drive passive wheels	To seamlessly initiate jumping
	Tricycle wheeling	Morphology transformation + Forward thrust propels passive wheels	Fast and efficient motion on flat terrain
	Object transfer	Synchronous paddling motion (low-frequency)	Delivery tasks of small-scale objects or when paths are blocked
Aquatic	Synchronous paddling	Paddling motion	Delicate motions in aquatic regions
	Air-propelled drifting	Forward air thrust force	Efficiency in aquatic regions
Aerial	Quadcopter flight	Cascaded PID attitude control	Long-distance transportation

**Table 2:** Dimensions of the robotic jumping mechanism.

Parameters	*a*	*b*	*c*	*d*	*e*	*l_1_*	*l_2_*	*l_3_*
Dimensions (m)	0.014	0.02065	0.025	0.025	0.01245	0.12	0.175	0.160

**Table 3:** PID controller gains.

Settings	K_P_	K_I_	K_D_
Roll rate	0.150	0.150	0.0020
Pitch rate	0.150	0.150	0.0020
Yaw rate	0.20	0.14	-
Roll angle	8	-	-
Pitch angle	6	-	-
Yaw angle	5	-	-

Note: The PID rate controller uses the parallel form. The unit for the roll, pitch, and yaw rate controller error is in rad/s, and the angle controller gives control output of angular velocity in rad/s for every 1 rad of angle error.

**Table 4:** Statistics of the average velocity, power, and COT of water-paddling-based swimming under five different groups of paddling frequencies.

	Frequency (Hz)	Trial 1	Trial 2	Trial 3	Trial 4	Trial 5	Mean	Standard Deviation
Velocity (m/s)	0.25	0.0770	0.0753	0.0760	0.0759	0.0791	0.0766	0.0015
	0.5	0.1667	0.1617	0.1643	0.1753	0.1657	0.1667	0.0051
	1	0.2923	0.2929	0.2915	0.2917	0.2947	0.2926	0.0013
	1.5	0.2765	0.3067	0.3193	0.3144	0.3156	0.3065	0.0174
	2	0.2988	0.2441	0.2568	0.2415	0.2504	0.2583	0.0234
Power (W)	0.25	2.4531	2.2898	1.9859	1.8187	1.9445	2.0984	0.2632
	0.5	3.0156	3.0881	2.9325	2.9640	2.8877	2.9779	0.0774
	1	5.2520	4.8359	4.9783	5.2088	5.3426	5.1235	0.2095
	1.5	7.7084	7.2885	6.8253	7.0312	6.8162	7.1339	0.3744
	2	8.5947	8.9848	8.8965	8.8226	8.5199	8.7637	0.1987
COT	0.25	8.8489	8.4463	7.2579	6.6556	6.8280	7.6073	0.9850
	0.5	5.0246	5.3045	4.9575	4.6964	4.8405	4.9647	0.2272
	1	4.9907	4.5859	4.7436	4.9598	5.0354	4.8631	0.1914
	1.5	7.7434	6.6007	5.9373	6.2117	5.9989	6.4984	0.7428
	2	7.9894	10.2236	9.6225	10.1472	9.4507	9.4867	0.9001

**Table 5:** Statistics of the average velocity, power, and COT of water-paddling-based swimming with different paddling spans.

	Span (cm)	Trial 1	Trial 2	Trial 3	Trial 4	Trial 5	Mean	Standard Deviation
Velocity (m/s)	40	0.2109	0.2143	0.2135	0.2158	0.2191	0.2147	0.0030
	45	0.2394	0.2503	0.2416	0.2488	0.2365	0.2433	0.0030
	50	0.2923	0.2929	0.2915	0.2917	0.2947	0.2926	0.0013
	55	0.2933	0.2691	0.2896	0.2882	0.2896	0.2860	0.0096
Power (W)	40	3.5902	3.2897	2.9904	3.7332	3.4426	3.4092	0.2866
	45	4.1841	4.1584	3.8069	3.9864	4.2929	4.0857	0.1907
	50	5.2520	4.8359	4.9783	5.2088	5.3426	5.1235	0.2095
	55	8.1304	7.9619	7.9591	8.2818	7.8744	8.0415	0.1633
COT	40	4.7283	4.2638	3.8904	4.8050	4.3642	4.4104	0.3711
	45	4.8545	4.6146	4.3766	4.4504	5.0418	4.6676	0.2782
	50	4.9907	4.5859	4.7436	4.9598	5.0354	4.8631	0.1914
	55	7.6995	8.2180	7.6336	7.9817	7.5524	7.8171	0.2763

**Table 6:** Statistics of the average velocity, power, and COT of the air-propelled drifting method with different levels of thrust commands.

	Thrust Command (%)	Trial 1	Trial 2	Trial 3	Trial 4	Trial 5	Mean	Standard Deviation
Velocity (m/s)	40	0.2166	0.2036	0.2142	0.1801	0.1758	0.1981	0.0191
	45	0.3227	0.3360	0.3234	0.3750	0.4201	0.3554	0.0420
	50	0.4468	0.4207	0.4064	0.4788	0.4530	0.4411	0.0284
	55	0.4960	0.4785	0.4833	0.4699	0.5051	0.4866	0.0140
Power (W)	40	1.6699	1.7744	1.6407	1.2416	1.2584	1.5170	0.2488
	45	4.3260	4.8352	4.6638	5.7431	7.2159	5.3568	1.1641
	50	8.5736	7.4197	7.1378	10.2607	8.8451	8.4474	1.2483
	55	12.7753	12.0593	12.2610	12.4449	14.1319	12.7345	0.8244
COT(non-accelerating phase)	40	2.0079	2.2905	2.2909	1.9009	1.8493	2.0679	0.2113
	45	4.4080	4.5092	4.3264	4.9282	5.8075	4.7959	0.6111
	50	6.5132	6.1372	5.7991	7.5755	7.2102	6.6470	0.7376
	55	8.9585	8.1117	8.1481	8.5955	9.3282	8.6284	0.5238

**Table 7:** Comparison of the structural reuse level of the proposed robot to some existing multi-modal platforms with aerial and terrestrial locomotion mechanisms.

Prototype	Reused Structure	
	Aerial Unit	Terrestrial Unit
Leonardo [39]	Thruster/High/IV	Leg/High/III
M4 [26]	Thruster/High/III	Leg/High/IV; Wheel/High/IV
MultiMo-Bat [9]	Fixed wing/Low/I	Jumping linkage/Medium/II
EPFL RAVEN [40]	Fixed wing/low/I; Thruster/low/I	Leg/High/III
Flying STAR [27]	Thruster/Medium/II	Wheel/Low/I
UIUC flying squirrel [41]	Gliding membrane/Medium/II	Leg/High/III
This work	Thruster/High/V	Leg/High/IV

Note: the structural reuse is classified into three levels: High, deployed three or more times; Medium, deployed twice; and Low, deployed once. The Roman numerals correspond to the total number of deployments of a unit. Here, the structural use of a unit requires its active activation to enable the operation of a mode.

## Data Availability

All data are available upon request.

## Supplementary Materials

The following supporting information can be downloaded at: https://www.mdpi.com/article/10.3390/biomimetics10060374/s1, Supplementary Videos: Video S1: Demonstration of the terrestrial locomotion modes; Video S2: Comparison of crawling on various terrains and crawling over a 6-cm-high obstacle; Video S3: Object transfer tasks; Video S4: Aquatic swimming tests. Supplementary information.pdf.
