# Supplementary material for: A Bipedal Robotic Platform Leveraging Reconfigurable Locomotion Policies for Terrestrial, Aquatic, and Aerial Mobility
Source: Biomimetics (Basel). 2025 Jun 5;10(6):374. doi: 10.3390/biomimetics10060374 (PMC12191364; doi:10.3390/biomimetics10060374)
Supplement: Supplementary file 1 [file biomimetics-10-00374-s001.zip › Supplementary information.pdf]

Supplementary information

S1. Control signal for the synchronous paddling motions

Both the synchronous crawling mode and the paddling-based swimming method use the same cyclical trigonometrical control signals described by formula (S1-S4):

$$\theta_{LS,1} = \begin{cases} -20 + \dfrac{\theta_H}{2}\cos\left(\dfrac{2\pi}{T}t\right) - \dfrac{\theta_H}{2}, 0 < t \le T \\ -\left(\dfrac{20}{\theta_H} - k\right)\theta_H - k\theta_H\cos\left(\dfrac{2\pi}{T}t\right), T < t \le 2T \\ recycle, t > 2T \end{cases}, \quad \text{(S1)}$$

$$\theta_{HS,1} = \begin{cases} -\dfrac{\theta_{S1}}{2}\cos\left(\dfrac{\pi}{T}t\right) + \dfrac{\theta_{S1}}{2}, 0 < t \le T \\ -\theta_{S1}\cos\left(\dfrac{\pi}{T}t\right), t > T \end{cases}, \quad \text{(S2)}$$

$$\theta_{LS,2} = \begin{cases} 20 - \dfrac{\theta_H}{2}\cos\left(\dfrac{2\pi}{T}t\right) + \dfrac{\theta_H}{2}, 0 < t \le T \\ \left(\dfrac{20}{\theta_H} - k\right)\theta_H + k\theta_H\cos\left(\dfrac{2\pi}{T}t\right), T < t \le 2T \\ recycle, t > 2T \end{cases}, \quad \text{(S3)}$$

$$\theta_{HS,2} = \begin{cases} 0 < t \le T : \dfrac{\theta_{S2}}{2}\cos\left(\dfrac{\pi}{T}t\right) - \dfrac{\theta_{S2}}{2} \\ t > T : \theta_{S2}\cos\left(\dfrac{\pi}{T}t\right) \end{cases}, \quad \text{(S4)}$$

Here, $\theta_{HS,\mathrm{i}}, \theta_{LS,i}$ (i is the leg number) are the hip and leg servo angle commands, with initial position defined in **Figure 7**. T corresponds to the time duration for a half of the paddling cycle, i.e., the paddling frequency is $1/(2T)$. θ_H, θ_S are predefined parameters that controls paddling height and span, and k is a

ratio parameter that independently sets the lift angle.

S2. Supplementary formulas for the modelling of crawling motions

***S2.1** The explicit expression of the side-view radius R_i regarding servo angle θ_{LS},θ_{HS}.*

The sideview radius R_i is calculated by formula (14) in the main text, based on the law of cosines. In formula (14), p' and l'_{hip} are the side-view projection of p and l_{hip}, and γ_1 is the angle between p' and the vertical line, as illustrated in **Figure 7B**, here:

$$p' = \sqrt{(p\sin(\pi-\theta_{LS}-\gamma_0)\sin\theta_S)^2 + (p\cos(\pi-\theta_{LS}-\gamma_0))^2}\,, \qquad \text{(S5)}$$

$$l'_{hip} = l_{hip}\sin\theta_{HS}\,, \qquad \text{(S6)}$$

$$\gamma_1 = \arccos(\frac{p\cos(\pi-\theta_{LS}-\gamma_0)}{p'})\,, \qquad \text{(S7)}$$

$\gamma_0 = 117.6^{\circ}$ is a fixed angle when the four-bar linkage leg is fully contracted, as shown in **Figure 7C.** The explicit expression of R_i with respect to θ_{LS},θ_{HS}, i.e., $R_i(\theta_{LS},\theta_{HS})$, can be obtained by substituting p', l'_{hip}, γ_1 into R_i:

$$R_i(\theta_{HS},\theta_{LS}) = \sqrt{\begin{array}{l} l_{hip}^2\sin^2\theta_{HS} + (p\sin(\pi-\theta_{LS}-\gamma_0)\sin\theta_S)^2 + (p\cos(\pi-\theta_{LS}-\gamma_0))^2 - \\ 2l_{hip}\sin(\theta_{HS})\sqrt{(p\sin(\pi-\theta_{LS}-\gamma_0)\sin\theta_S)^2 + (p\cos(\pi-\theta_{LS}-\gamma_0))^2} \\ \times\cos(\arccos(\frac{p\cos(\pi-\theta_{LS}-\gamma_0)}{p}) + \pi/2) \end{array}}\,, \qquad \text{(S8)}$$

***S2.2.** Calculation of the inclination angle*

The inclination angle of the robot's body frame θ_{inc} (as shown in **Figure 7A**) is obtained via geometrical calculation (by sine and cosine laws):

$$\theta_{inc} = \arcsin\left(\frac{\sin(\phi_{cr}-\varepsilon)(R_i+r_2)}{\sqrt{(t+w/2)^2+s^2+(R_i+r_2)^2+2\sqrt{(t+w/2)^2+s^2}\bullet(R_i+r_2)\cos(\phi_{cr}-\varepsilon)}}\right)-\varepsilon, \quad \text{(S9)}$$

where:

$$\varepsilon = \arcsin\left(\frac{s}{\sqrt{(t+w/2)^2+s^2}}\right), \quad \text{(S10)}$$

$$\phi_{cr} = \arcsin\left(\frac{l'_{hip}\ \sin(\gamma_1+\pi/2)}{R_i}\right)+\gamma_1+\pi/2, \quad \text{(S11)}$$

By substituting (S6), (S7), (S8), (S10), and (S11) into (S9), the explicit form of inclination angle θ_{inc} can be obtained.

S3. Cost of transport for the crawling mode

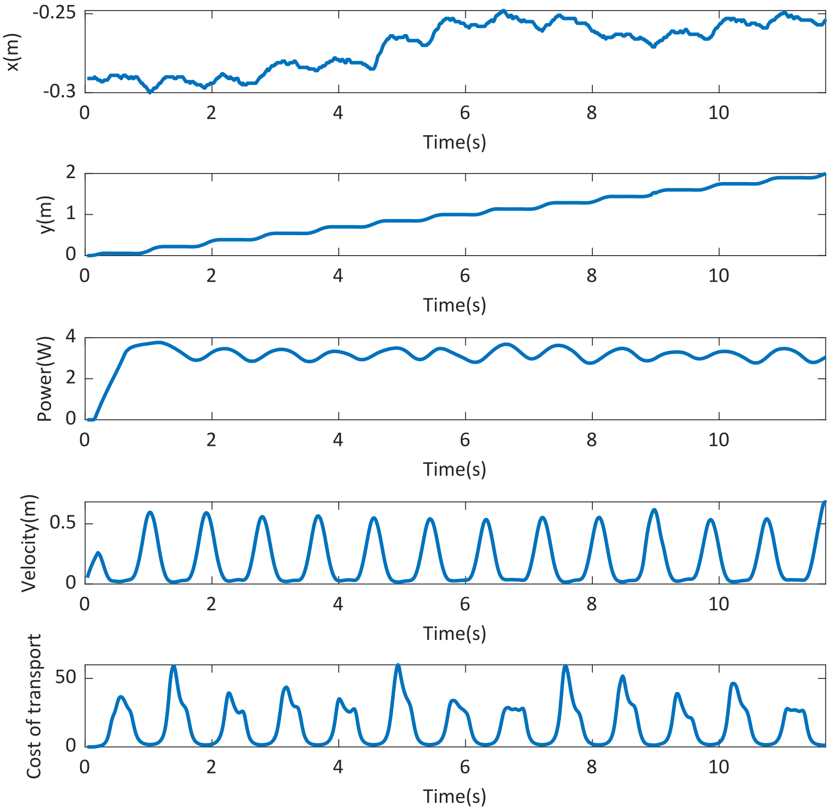

Figure S1. Demonstration of the crawling speed, power and instantaneous cost of transport.

Figure S1 presents the instantaneous crawling speed, power consumption and the cost of transport. The planar translation energy efficiency index, i.e., the average cost of transport, is calculated by:

$$COT_{avg} = \frac{\overline{P}}{mg\overline{v}} = \frac{3.14}{0.367 \times 9.81 \times 0.171} = 5.11, \quad \text{(S12)}$$

The test was conducted on the flat ground shown in **Figure 15**. The power was read back wirelessly from a voltage and current sensor module. The motion was video recorded at 60 FPS and the velocity is analyzed by a video analysis tool (Tracker).

S4 Energy consumption in the jumping-assistive flight

During the jumping-assistive flight test, the voltage during the flight was measured using the voltage module as used in the swimming test, and wirelessly sent back to the laptop. However, due to distance and electric noise, the streamed data contains messy characters. Here we use another method which measures the energy consumed to discharge the battery from (4S 300 mAh, Gaoneng Battery (Dongguan) Co. Ltd, China) fully charged to the level at the apex (16.23 V) using a battery charger (ToolkitRC M6, ToolkitRC Technology (Shenzhen) CO.,Ltd, China) with a discharge current of 0.5 A. The discharge curve is shown in **Figure S2A** and total discharged electric charge is 65 mAh (**Figure S2B**). The total energy is calculated by integrating the curve in **Figure S2A**, i.e., $E_{discharge} = \sum_i U_i Q_i$.

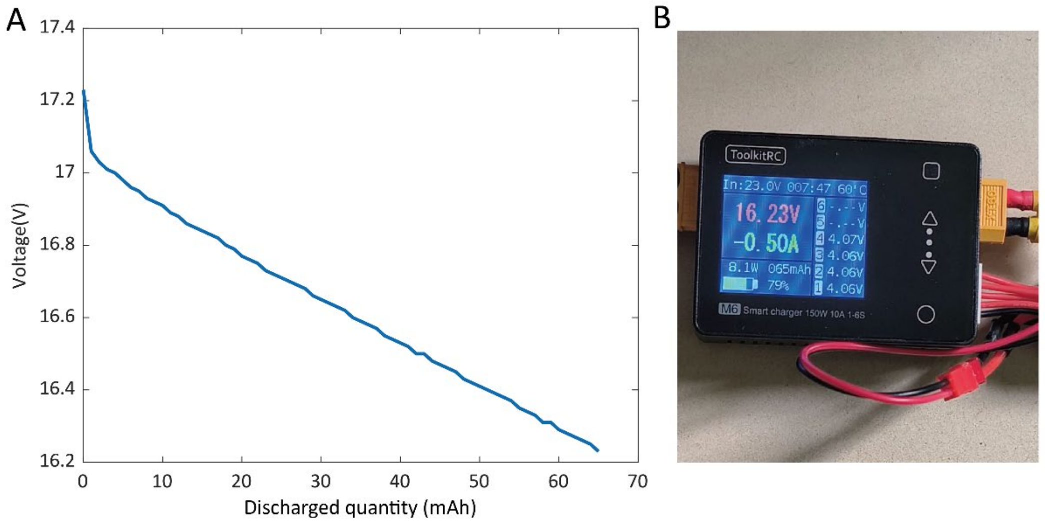

Figure S2. Energy estimation via measuring discharged electric charge. A. Discharge curve between voltage level and quantity of electric charge. **B**. Demonstration of the total discharged electric charge (65mAh).
